# Modulation of Hepatic Insulin and Glucagon Signaling by Nutritional Factors in Broiler Chicken

**DOI:** 10.3390/vetsci9030103

**Published:** 2022-02-25

**Authors:** Janka Petrilla, Gábor Mátis, Máté Mackei, Anna Kulcsár, Csilla Sebők, Márton Papp, Péter Gálfi, Hedvig Fébel, Korinna Huber, Zsuzsanna Neogrády

**Affiliations:** 1 Division of Biochemistry, Department of Physiology and Biochemistry, University of Veterinary Medicine, 1078 Budapest, Hungary; matis.gabor@univet.hu (G.M.); mackei.mate@univet.hu (M.M.); qpanka@gmail.com (A.K.); sebok.csilla@univet.hu (C.S.); neogrady.zsuzsanna@univet.hu (Z.N.); 2 Center for Bioinformatics, University of Veterinary Medicine, 1078 Budapest, Hungary; pappmarci95@gmail.com; 3 Department of Pharmacology and Toxicology, University of Veterinary Medicine, 1078 Budapest, Hungary; galfi.peter@univet.hu; 4 Nutrition Physiology Research Group, Institute of Physiology and Nutrition, Kaposvár Campus, Hungarian University of Agriculture and Life Sciences, 2053 Herceghalom, Hungary; hullarne.febel.hedvig@uni-mate.hu; 5 Department of Functional Anatomy of Livestock, Institute of Animal Science, University of Hohenheim, 70593 Stuttgart, Germany; korinna.huber@uni-hohenheim.de

**Keywords:** insulin, insulin receptor beta, glucagon, hepatic glucagon receptor, mammalian target of rapamycin, butyrate, non-starch polysaccharides, chicken

## Abstract

Influencing the endocrine metabolic regulation of chickens by nutritional factors might provide novel possibilities for improving animal health and productivity. This study was designed to evaluate the impact of dietary cereal type (wheat-based (WB) vs. maize-based (MB) diets), crude protein level (normal (NP) vs. lowered (LP)), and sodium (n-)butyrate (1.5 g/kg diet) supplementation (vs. no butyrate) on the responsiveness of hepatic glucagon receptor (GCGR), insulin receptor beta (IRβ) and mammalian target of rapamycin (mTOR) in the phase of intensive growth of chickens. Liver samples of Ross 308 broiler chickens (*Gallus gallus domesticus*) were collected on day 21 for quantitative real-time polymerase chain reaction and Western blot analyses. Hepatic GCGR and mTOR gene expressions were up-regulated by WB and LP diet. GCGR and IRβ protein level decreased in groups with butyrate supplementation; however, the quantity of IRβ and mTOR protein increased in WB groups. Based on these data, the applied dietary strategies may be useful tools to modulate hepatic insulin and glucagon signaling of chickens in the period of intensive growth. The obtained results might contribute to the better understanding of glycemic control of birds and increase the opportunity of ameliorating insulin sensitivity, hence, improving the production parameters and the welfare of broilers.

## 1. Introduction

Intensive growth as well as animal welfare is of special importance in broiler meat production, which is commonly promoted by the application of short-chain fatty acids (SCFA; [1]), and particularly that of the four-carbon butyric acid (butyrate; [2]). Butyrate can be used as feed supplement (exogenous origin), or produced endogenously by anaerobe microbial fermentation of carbohydrates in the ceca of broilers [3]. The formation of SCFA can be enhanced by the application of ingredients with an elevated ratio of soluble non-starch polysaccharides (NSP) in the feedstuff, such as rye or wheat [4,5], the latter containing more NSP compared to maize [4,6], mostly arabinoxylans [7]. The molar ratio of acetate, propionate and butyrate in the ceca varies from approximately 75:15:10 to 40:40:20 in a healthy chicken [8]. Although butyrate is present in the lowest proportion amongst the mentioned SCFA, it has notable effects on the resident microbiota and on the host itself [9,10]. In addition to the gut, the liver is the organ with the highest exposure to the absorbed SCFA and so to the biologically most active butyrate [11].

Butyrate has the ability to alter gene expression epigenetically [12,13,14,15,16], and receptor-mediated effects have also been reported [17]. These properties might lead to the modulation of metabolic pathways and the hormone system, affecting the productivity and health of the animals.

Similarly to mammals, the carbohydrate metabolism of birds is primarily regulated by insulin and by its antagonist, glucagon [18], both activating intracellular pathways after binding to their receptors. The mammalian target of rapamycin (mTOR) is one of the insulin-activated downstream elements and known to be the cardinal stimulator of muscle growth via protein synthesis [19]. Although insulin sensitivity of avian tissues is significantly lower compared to mammals [20], insulin is involved in the carbohydrate and lipid metabolism, as well as in the growth of the animals. On the other hand, glucagon is responsible for the regulation of glycogen metabolism and gluconeogenesis, and due to the high glucagon sensitivity of the liver, this hormone plays a key role in the carbohydrate metabolism of this organ [18,21].

Orally administered butyrate proved to affect the insulin signaling of broilers on a tissue-dependent manner as a daily oral bolus [22]. The potential of a fiber-rich diet to influence insulin homeostasis has also been declared in different species [6,23,24].

Another alternative solution to optimize the growth of broilers is a reduction in crude protein (CP) content of diets. This method has a huge economic and environmental impact, providing a possibility for decreased nitrogen excretion [25], while a moderate reduction in dietary CP content with limiting amino acid supplementation does not result in growth depression of chicken [26], but is able to alter a chemical composition of the meat [27,28]. However, it is still poorly investigated how this special feeding condition (moderately reduced dietary CP level with limiting amino acid completion) affects the endocrine system and, thus, the carbohydrate metabolism of broilers.

The hypothesis of our study was that, similar to other observations, [6] exogenous- (feed additive sodium [n-]butyrate) and endogenously produced (anaerobe microbial fermentation product of NSP) butyrate might affect the metabolism of chicken differently—primarily through insulin and glucagon signaling—or have a different action when applied in combination.

Accordingly, we examined the responsiveness of three key members of insulin and glucagon signaling in the liver—glucagon receptor (GCGR), insulin receptor beta (IRβ) and mammalian target of rapamycin (mTOR)—in the phase of intensive growth (1) to different, widely used cereal types (wheat vs. maize, as cereal grains differing in soluble NSP content), (2) to various dietary CP levels (normal vs. reduced CP content of the diet, the latter with limiting amino acid supplementation) and (3) to butyrate as a feed additive, on both mRNA and protein level, in order to gain deeper knowledge on the biochemical and physiological background of the actions and interactions of these above-mentioned dietary factors in broiler chicken.

## 2. Materials and Methods

### 2.1. Animals

Eighty newly hatched male Ross 308 broiler chicks (*Gallus gallus domesticus*) were purchased from a commercial hatchery (Gallus Company, Devecser, Hungary) and were randomly classified to eight dietary groups (*n* = 10 per group), each showing similar average body weights. Environmental conditions met the recommendations of Ross technology [29]. Upon arrival, the animals were housed on wheat straw litter in floor pens (2.54 × 1.85 m each), with the whole house temperature set to 30 °C. The temperature was then decreased to 28 °C by decreasing it with 1 °C/day, later lowered by 1 °C/3 days until 22 °C was reached on day 21. The relative humidity was set between 60–70% on the first 3 days after placement, then kept above 50% throughout the remaining part of the experimental period (controlled daily with a hygrometer). The illumination was ensured with incandescent bulbs of warm white color. The light intensity was set to 30–40 lux in the broiler house with 23 h light and 1 h dark period during the first week of life, then lowered to 10 lux with 20 h light and 4 h continuous dark period until day 21. Feed and drinking water were available *ad libitum*. Animals were monitored daily and the growth performance of the birds matched the parameters detailed in the Broiler Management Handbook: Ross 308 [29].

Dietary treatments followed a 2 × 2 × 2 factorial arrangement, forming eight dietary groups, as follows: The applied diets were maize- or wheat-based (maize-based (MB) or wheat-based (WB) diet), with or without sodium butyrate supplementation in the commonly used dose in poultry nutrition (1.5 g/kg diet, Sigma-Aldrich, Budapest, Hungary), which was already successfully applied in our earlier studies [6,30]. Furthermore, all diets were designed with CP content matching standard recommendations of the adequate dietary phase (“normal protein” (NP) groups with 22.7% and 21.4% CP in starter and grower diets) or reduced by 15% (“low protein” (LP) groups with 19.1% and 18.0% CP, respectively). The most important limiting amino acids (L-lysine, DL-methionine, L-threonine and L-tryptophan) were supplemented to “LP” diets to meet the recommendations of the breeder [29]. Starter diets were switched to grower diets on day 10. All diets were set isoenergetic within a phase, designed to suit the nutrient specifications of Ross 308 recommendations [29] and fed in mash form. The compositions and calculated nutrient contents of diets (without sodium [n-]butyrate supplementation) are indicated in Table 1 and Table 2. All the diets were formulated and produced by the feed mixing facility of the Hungarian University of Agriculture and Life Sciences (Herceghalom, Hungary).

### 2.2. Sampling

Body weights of the birds were measured individually on 7 d, 14 d and 21 d with a digital scale of 0.1 g accuracy to gain background data; then, on 21 d, the animals were decapitated in CO_2_ narcosis, the liver was excised and tissue samples were obtained for quantitative real-time polymerase chain reaction (qRT-PCR) and Western blot analyses. PCR samples were taken and placed into RNA isolation reagent (easy-BLUE^TM^, Sigma-Aldrich) and placed on dry ice, while Western blot samples were shock-frozen in liquid nitrogen, then all the samples were stored at −80 °C until further processing.

### 2.3. qRT-PCR Measurements

Reagents were obtained from Reanal (Budapest, Hungary), except when otherwise specified.

After thawing on ice, tissue samples were aseptically homogenized by a tissue grinder Potter-Elvehjem homogenizer, centrifuged (Beckman-Coulter, Indianapolis, IN, USA; 12,000× *g*, 10 min, 4 °C), then the middle homogenous phase was centrifuged again (12,000× *g*, 10 min, 4 °C). After incubation of the clean supernatant for 5 min at room temperature, 200 µL chloroform was measured into the tubes followed by vigorous shaking, then another incubation for 5 min at room temperature was applied and samples were centrifuged (13,000× *g*, 10 min, 4 °C) for phase separation. Thereafter, aqueous top phase was mixed with an equal amount of isopropanol, shaken and incubated for 10 min at room temperature and centrifuged (13,000× *g*, 5 min, 4 °C). After that, 1 mL of 75% ethanol was measured onto the RNA pellet, followed by subsequent centrifugation (10,000× *g*, 5 min, 4 °C). As a next step, the pellet was dried for 10 min at 60 °C, then dissolved in 50 µL molecular biology grade water, incubated for 10 min at 60 °C and finally placed on ice. Removal of genomic DNA was performed with RapidOut DNA Removal Kit (Biocenter, Szeged, Hungary; catalogue number: K2981), synthesis of cDNA was implemented with RevertAid First Strand cDNA Synthesis Kit (Biocenter, Szeged, Hungary; catalogue number: K1622), according to the instructions of the manufacturer. qRT-PCR measurement was performed with a Rotor-Gene Q thermocycler (Qiagen, Hilden, Germany; software version 2.1.0, Build 9) and applying Thermo Scientific Luminaris Color HiGreen qPCR Master Mix (Biocenter, Szeged, Hungary). Primer pairs were designed with NCBI Primer-BLAST and purchased from Biocenter (Szeged, Hungary), applied as detailed in Table 3 to test genes of interest. Glyceraldehyde-3-phosphate dehydrogenase (GAPDH) was chosen as the housekeeping gene; its expression was unaffected by any of the applied dietary treatments. The temperature profile of the reaction was set as follows: uracil-DNA glycosylase treatment at 50 °C for 2 min; initial denaturation at 95 °C for 10 min; denaturation step at 95 °C for 15 s; annealing at 60 °C for 30 s and extension at 72 °C for 30 s for 40 cycles. At the end of each cycle, fluorescence monitoring was set for 10 s. Relative gene expressions were calculated by the 2^–∆∆Ct^ method applying the software 2.1.0 (Build 9, Qiagen, Hilden, Germany)

### 2.4. Western Blot Measurements

The protein abundance of GCGR, IRβ and mTOR was determined from liver samples by semiquantitative Western blotting in duplicates. Western blot measurements were implemented at the University of Hohenheim, Institute of Animal Science (Hohenheim, Germany). Reagents were purchased from Sigma-Aldrich (Munich, Germany), except when otherwise specified.

Approximately 300 mg of liver sample was completed with 0.6 mL lysis buffer as described by Kenéz et al. [31], homogenized for 40 s using FastPrep^®^-24 Classic homogenizer (MP Biomedicals, Santa Ana, CA, USA), then centrifuged (1000× *g*, 5 min, 4 °C). Protein concentration of supernatants was measured by colorimetric method with Bradford reagent (Serva Electrophoresis GmbH, Heidelberg, Germany), then samples were diluted to an equal (1.5 μg/μL) protein concentration with loading buffer [31] and processed with heat denaturation (5 min, 95 °C). Electrophoresis was performed in duplicates in 5% stacking (60 V, 30 min) and 8.1% separation (120 V, 90 min) polyacrylamide gel (20 μL loading volume per lane). After tank blotting (25 V, 20 min), membranes were blocked in 5% BSA containing phosphate-buffered saline with Tween 20 (PBST; 60 min, room temperature), followed by overnight incubation at 4 °C with primary antibodies (diluted with 5% BSA/PBST) in the following concentrations: 1:500 for GCGR (Santa Cruz Biotechnology, Paso Robles, CA, USA); 1:3000 for IRβ (Cell Signaling Technology, Frankfurt, Germany) and 1:2100 for mTOR (Santa Cruz Biotechnology, Paso Robles, CA, USA), tested by Mátis et al. [22]. Detection of primary antibodies was performed using an anti-rabbit secondary antibody coupled with horseradish peroxidase (Cell Signaling Technology, Frankfurt, Germany), in the concentrations as 1:3000 dilution in 5% BSA/PBST for GCGR, and 1:2500 dilution in 2.5% BSA/PBST for IRβ and mTOR (60 min, room temperature). Finally, chemiluminescence was generated with the SuperSignal West Dura Extended Duration Substrate (Pierce, Rockford, IL, USA) and detected with ChemiDoc XRS+ system (Bio-Rad Laboratories GmbH, Feldkirchen, Germany.). Bands were quantified by densitometry using Image Lab 4.0 software; relative protein abundance values were gained by standardizing trace quantities to the Indian Ink-stained bands.

### 2.5. Statistical Analyses

Statistical analyses of data were carried out with R 4.0.3. software. Data were plotted as boxplots for exploratory analysis. Three independent linear models were fitted to every measured variable (GCGR, IRβ and mTOR as outcome variables) based on the following model:

(GCGR, IRβ, mTOR) ≈ Cereal + Protein + Butyrate + Cereal* Protein + Cereal* Butyrate + Protein* Butyrate + Cereal* Protein* Butyrate

Model fitting was performed with the lm built-in function. Statistical significances of the main and interaction effects were evaluated by a multivariate ANOVA analysis, using the ANOVA function of the car package. The R package emmeans was used to perform pairwise comparison of the estimated marginal means to unravel the differences behind the different treatment groups. *p*-values and confidence levels were adjusted with the Tukey method. Results were considered statistically significant when *p* < 0.05.

## 3. Results

In this section, only significant effects of the investigated dietary factors are presented in detail.

### 3.1. Glucagon Receptor

Multivariate ANOVA analysis showed that GCGR gene expression was significantly increased in WB compared to MB groups (*p* = 0.0007), and in chickens kept on LP diet in contrast to animals of the NP groups (*p* = 0.0067; for more details, see Tables S1 and S2). Pairwise comparisons revealed significant differences between WB LP Ctr vs. MB NP Ctr (*p* = 0.0083), MB NP But (*p* = 0.0150) and WB NP Ctr groups (*p* = 0.0221). More details on the pairwise comparisons can be observed in Table S3.

On the protein level, significant decreasing effect of butyrate (*p* = 0.0224) and the interactions of the dietary cereal type and butyrate supplementation (*p* = 0.0226), as well as CP content and butyrate supplementation (*p* = 0.0172), were observed (Tables S1 and S2). Pairwise comparisons showed significant differences between WB NP Ctr vs. MB LP Ctr (*p* = 0.0157), MB NP But (*p* = 0.0057), WB LP But (*p* = 0.0221) and WB NP But (*p* = 0.0212) groups. For further details on the pairwise comparisons, see Table S4.

**Figure 1:**
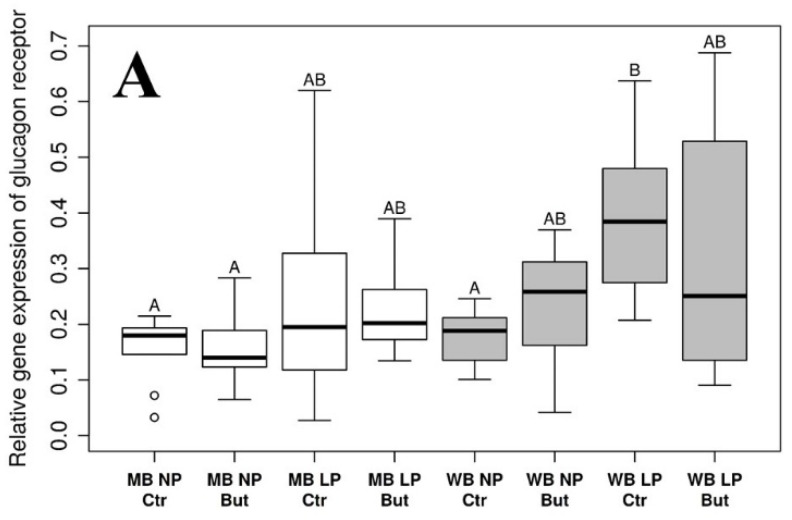

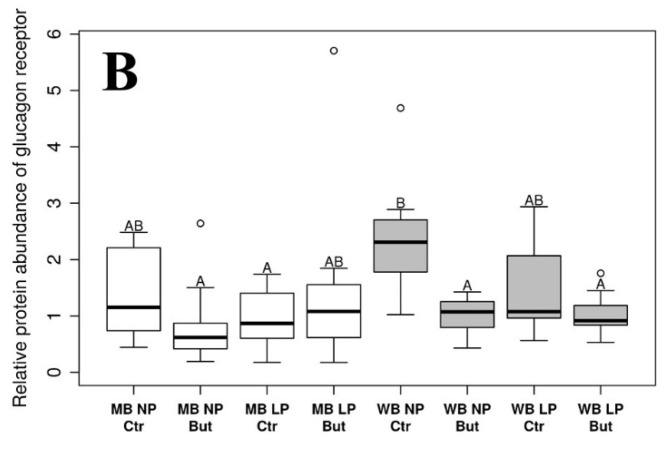
Relative gene expression (**A**) and protein abundance (**B**) of hepatic glucagon receptor. MB: maize-based diet; WB: wheat-based diet supplemented with NSP-degrading xylanase and glucanase enzymes; NP: “Normal protein” group reared on a diet with crude protein content adequate to the dietary phase; LP: “Low protein” group reared on a diet with crude protein content reduced by 15%, supplemented with limiting amino acids; But: sodium butyrate supplementation of the diet in the dose of 1.5 g/kg diet; Ctr: control group without sodium butyrate supplementation. Letters indicate groups that were significantly different with pairwise comparison. If two groups have different letters, *p*-values were <0.05.

Diet-induced changes in GCGR gene expression and protein abundance with the results of pairwise comparisons can be seen on Figure 1A,B.

Statistical analysis of data was performed by a multivariate ANOVA test to evaluate the main effects, and pairwise comparisons were implemented by the *emmeans* R package with *p* value adjustments with the Tukey method. Empty bars refer to MB and grey bars refer to WB groups. *n* = 10 per group.

### 3.2. Insulin Receptor β

A significant three-way interaction effect between all dependent variables was detected with ANOVA analysis in case of IRβ mRNA concentration (*p* = 0.0028; Tables S1 and S2). Pairwise comparisons revealed significant differences between WB LP Ctr vs. WB NP Ctr (*p* = 0.0226) and MB NP But (*p* = 0.0270) groups. Table S5 shows additional details on the pairwise comparisons.

A significant lowering effect of butyrate (*p* = 0.0343) and an augmenting effect of the WB diet (*p* < 0.0001) were detected as main effects. Furthermore, significant cereal:protein (*p* = 0.0006) and protein:butyrate (*p* = 0.0007) interactions were found by the ANOVA analysis of the IRβ Western blot results, referring to protein abundance (Tables S1 and S2). According to the pairwise comparisons, either WB LP But, WB NP But and WB NP Ctr groups differed significantly from every MB group (*p*-values ranging from <0.0001 to 0.0132), while WB LP Ctr was significantly different only from the MB NP But treatment from the MB group (*p* = 0.0002). Considering the WB group solely, WB NP Ctr was significantly different from every other WB treatment (*p*-values ranging from 0.0002 to 0.0417), while no significant differences were detected in the MB groups. For more details, see Table S6.

**Figure 2:**
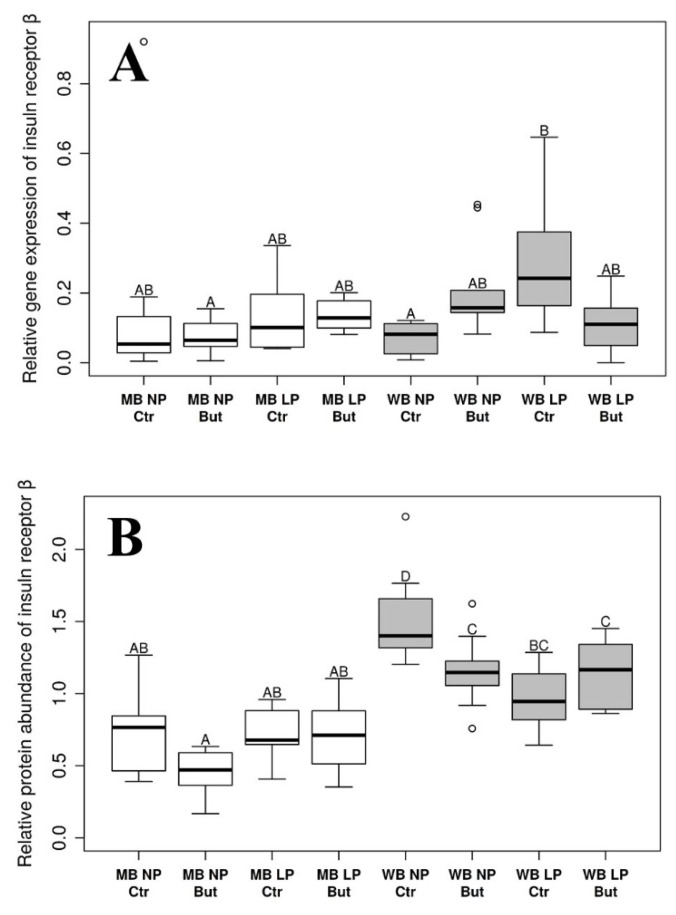
Relative gene expression (**A**) and protein abundance (**B**) of hepatic insulin receptor β. MB: maize based diet; WB: wheat based diet supplemented with NSP-degrading xylanase and glucanase enzymes; NP: “Normal protein” group reared on a diet with crude protein content adequate to the dietary phase; LP: “Low protein” group reared on a diet with crude protein content reduced by 15%, supplemented with limiting amino acids; But: sodium butyrate supplementation of the diet in the dose of 1.5 g/kg diet; Ctr: control group without sodium butyrate supplementation. Letters indicate groups that were significantly different with pairwise comparison. If two groups have different letters, *p*-values were <0.05.

Changes in IRβ gene expression and protein abundance, together with the results of pairwise comparisons, can be seen in Figure 2A,B.

Statistical analysis of data was performed by a multivariate ANOVA test to evaluate main effects, and pairwise comparisons were implemented by the *emmeans* R package with *p* value adjustments with the Tukey method. Empty bars refer to MB and grey bars refer to WB groups. *n* = 10 per group.

### 3.3. Mammalian Target of Rapamycin

The mTOR mRNA concentration showed a significant increase in chickens fed the WB diet in comparison with their counterparts on the MB diet (*p* = 0.0456) and was significantly augmented in LP group compared to animals fed the NP diet (*p* < 0.0001; Tables S1 and S2). Further, significant differences were found between the WB LP Ctr vs. MB NP But (*p* = 0.0131), MB NP Ctr (*p* = 0.0012), WB NP But (*p* = 0.0179) and WB NP Ctr treatment groups (*p* = 0.0094; Table S7).

Protein abundance of mTOR was significantly higher in animals fed the WB diet than in those kept on the MB diet (*p* = 0.001). Considering the pairwise comparisons, only the MB NP But and WB LP But groups differed significantly (*p* = 0.0199). More details can be seen in Tables S1, S2 and S8.

**Figure 3:**
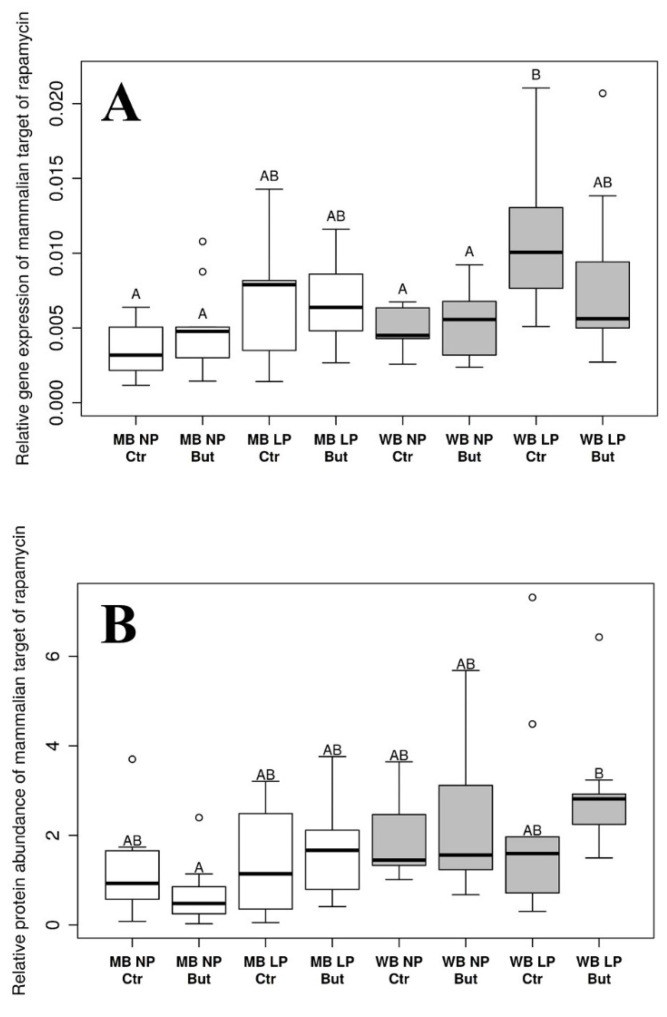
Relative gene expression (**A**) and protein abundance (**B**) of hepatic mammalian target of rapamycin. MB: maize-based diet; WB: wheat-based diet supplemented with NSP-degrading xylanase and glucanase enzymes; NP: “Normal protein” group reared on a diet with crude protein content adequate to the dietary phase; LP: “Low protein” group reared on a diet with crude protein content reduced by 15%, supplemented with limiting amino acids; But: sodium butyrate supplementation of the diet in the dose of 1.5 g/kg diet; Ctr: control group without sodium butyrate supplementation. Letters indicate groups that were significantly different with pairwise comparison. If two groups have different letters, *p*-values were <0.05.

For diet-related changes in mTOR gene expression and protein abundance, see Figure 3A,B.

Statistical analysis of data was performed by a multivariate ANOVA test to evaluate main effects, and pairwise comparisons were implemented by the emmeans R package with *p* value adjustments with the Tukey method. Empty bars refer to MB and grey bars refer to WB groups. *n* = 10 per group.

Representative bands obtained by Western blotting are presented in Table 4.

**Figure 4:**
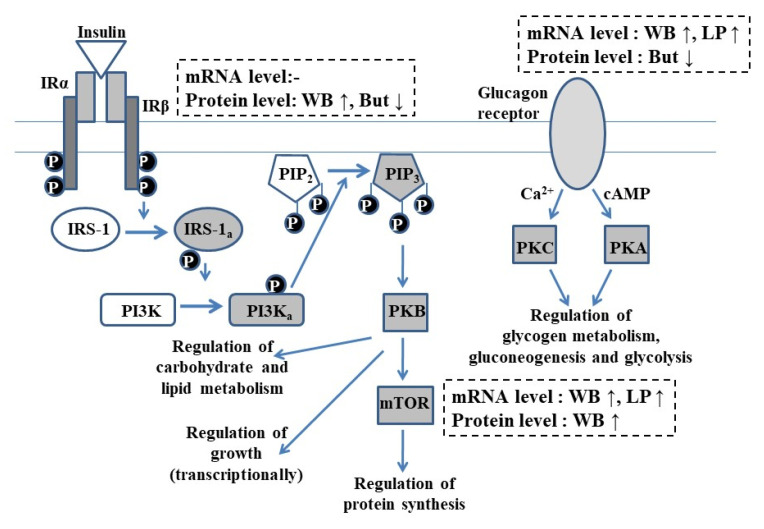
Overview of the insulin and glucagon signaling pathways and the observed main effects of the investigated nutritional factors. IRα: insulin receptor α subunit; IRβ: insulin receptor β subunit; IRS-1: insulin receptor substrate 1 (“a” lowercase subscript when activated); PI3K: phosphatidylinositol-3-kinase (“a” lowercase subscript when activated); PIP_2_: phosphatidylinositol diphosphate; PIP_3_: phosphatidylinositol triphosphate; PKB: protein kinase B; mTOR: mammalian target of rapamycin; Ca^2+^: calcium ion; cAMP: cyclic adenosine monophosphate; PKC: protein kinase C; PKA: protein kinase A; P: phosphate group; MB: maize-based diet; WB: wheat-based diet supplemented with NSP-degrading xylanase and glucanase enzymes; NP: “Normal protein” group reared on a diet with crude protein content adequate to the dietary phase; LP: “Low protein” group reared on a diet with crude protein content reduced by 15%, supplemented with limiting amino acids; But: sodium butyrate supplementation of the diet in the dose of 1.5 g/kg diet. ↓ and ↑ arrows indicate the lowering or increasing effect of the given nutritional factor on the investigated parameter.

The observed diet-associated main effects on insulin and glucagon signaling are summarized in Figure 4, also providing an overview of the signaling pathways.

Once insulin binds to the insulin receptor α subunit, the β subunit becomes autophosphorylated, activating PI3K and PIP_2_ by phosphorylation and, finally, the mammalian target of rapamycin via PKB. When glucagon binds to its G-protein-coupled glucagon receptor, the elevation in the intracellular cAMP and Ca^2+^ ion concentration leads to the activation of PKA and PKC, respectively.

## 4. Discussion

In this study, the conventional MB diet represents a lower level of soluble NSP, affecting cecal butyrate production to a lesser extent, compared to the WB diet. The soluble arabinoxylan content was previously measured as 0.88 mg/g for maize and 9.37 mg/g for wheat (determination performed in the Agricultural Institute, Center for Agricultural Research, Martonvásár, Hungary, following the method of Douglas [32]). A high arabinoxylan content of the diet was one of the most probable candidates to enhance intestinal SCFA production, especially that of butyrate, and to increase plasma butyrate level in several studies [7,33]. However, it should be taken into consideration that maize and wheat also differ in some other parameters (such as amino and fatty acid profiles); thus, the impact of certain further nutrients on the observed diet-associated changes cannot be excluded. The wheat-based diet was formulated with the inclusion of xylanase and glucanase NSP-degrading enzymes that cleave long, soluble NSP chains to shorter oligosaccharides [34], moderating the antinutritive effects of wheat [35]. Further, enzymatic cleavage enhances bacterial SCFA production [36], primarily that of butyrate, as detected in earlier trials [5,6,30], enabling the comparison of the effects associated with higher (WB) and lower (MB) cecal SCFA production levels. An application of sodium butyrate aimed the comparison of the effect of this widely used, exogenous butyrate source with that of an endogenously produced one.

The diet of LP groups was completed with limiting amino acids L-lysine, DL-methionine, L-threonine and L-tryptophan, in order to avoid the growth depression caused by inadequate amino acid supply. Therefore, the ratio of free limiting amino acids was higher in LP diets compared to NP groups, resulting in better bioavailability, thus, better absorbance of the above-mentioned amino acids [37].

The optimal age of birds for the investigation of the selected parameters was determined according to data from the literature. Previous studies showed that, independently from the technology and breed applied, the growth rate of is increases in the middle phase of the life of the animals [38,39]. An age-related decrease in the sensitivity of insulin signaling proteins to nutritional factors was also measured in mammals [40] and in birds [41,42], while the opposite was observed for glucagon sensitivity in chickens [41]. In summary of all the above-described processes, and taking into account the 6-week conventional rearing technology, the age of 3 weeks was considered optimal. In week 3, the metabolism of broilers is highly intensive with an increasing responsiveness to glucagon, but the sensitivity of insulin signaling elements might be still high enough; therefore, the most relevant information could be expected this age.

An increase in GCGR mRNA expression was observed in WB and LP groups (compared to chickens kept on MB and NP diet, respectively), but these changes could not be detected on a protein level. However, exogenous butyrate decreased GCGR content of the liver without observed alterations of GCGR mRNA production, and the interaction of dietary cereal type and butyrate supplementation—as well as CP content and butyrate supplementation—was also detected on a protein level. Pairwise comparison showed that GCGR exhibited higher protein levels with no butyrate supplementation in the NP group, and this effect was more pronounced when the animals were fed the WB diet. A previous trial showed that butyrate downregulated the GCGR protein abundance of chicken adipocytes in an in vitro study [43]. Furthermore, the origin (exogenous feed additive or endogenously produced in the intestines) and application form (feed supplementation or bolus) of butyrate could influence the mode of action of this molecule [6,22]. Although the epigenetic- and receptor-mediated effects of butyrate were not investigated in this study, one possible explanation for the apparent inconsistency of mRNA and protein levels might be attributed to the butyrate-evoked partial inhibition of the post-transcriptional processing of GCGR mRNA.

Concerning the expression data of IRβ and mTOR as prominent members of the intracellular insulin signaling pathway, IRβ mRNA concentration was significantly affected by the interaction of all dietary factors, while mTOR mRNA level increased in WB and LP groups. However, an increase in the protein level of both elements was observed in WB groups, while the butyrate supplementation of the diet decreased hepatic IRβ protein abundance, and the interaction of dietary cereal and CP content of the diet, as well as CP content and butyrate supplementation, was also detected. Interestingly, the WB diet could exert its IRβ protein abundance-inducing effect more when combined with NP; butyrate also had a more pronounced decreasing effect in the NP groups, suggesting that independent of the origin, butyrate was more potent in affecting hepatic IRβ protein abundance in the NP groups. Despite the higher CP content of NP diet, in certain aspects it can be considered as a diet with less favorable composition due to the lack of easily available limiting amino acids; it is conceivable that, similarly to other observations, butyrate has a more notable influence in suboptimal conditions [44].

Although the structure and function of insulin receptors in birds are similar to those of mammals [19], the physiological protein levels of both receptor subunits and insulin receptor substrate 1 (IRS-1)—as well as the inducibility of the insulin cascade mechanism—are lower in chickens than in mammalian species [45]. Because of this moderate responsiveness to insulin, the observed increase in IRβ and mTOR protein abundance in WB groups could be of outstanding importance in the activation of downstream elements of the signaling pathway, suggesting that there is an opportunity to find new ways of growth promotion in the broiler industry via the amelioration of insulin sensitivity. Notwithstanding that the present study was not designed to assess performance data, it should be noted as background information that the body weight of chickens was increased by applying WB and LP (limiting amino acid supplemented) diets, as published by Petrilla et al. [46]. These results are in accordance with those of previous studies [28,30], and might be partly related to the detected amelioration of the IRβ and mTOR protein abundance in WB groups.

It is highlighted by the present results that the type of cereal could exert modulatory action on IRβ and mTOR expression on both mRNA and protein level; however, this effect highly depended on the CP content of the applied diet in the case of IRβ. The increased amount of IRβ and mTOR protein in the liver might be a result of the enhanced microbial butyrate synthesis by the soluble NSP content of WB diet in the ceca. In this respect, our results are in line with the observation of Kulcsár et al. [6], who also found elevated hepatic IRβ and mTOR protein level in the animals of WB group at the age of 6 weeks. Endogenously produced SCFA and primarily butyrate was shown to increase insulin sensitivity and glycemic control in several in vivo studies with mammals [23,24]. Furthermore, Ramiah et al. [47] found an increased mTOR mRNA level in the liver of broiler chickens, noting the high NSP level of the feed as a possible explanation. In our previous paper, we already reported lowered blood glucose levels of the animals used in this experiment in the WB group at the age of 3 weeks [46]. However, we did not detect any change in the plasma insulin concentration of the WB group compared to the MB group [46]. This is in alignment with the results of Józefiak et al. [48], who reported increased insulin sensitivity of the liver of broiler chickens fed a wheat–soybean-based, enzyme-supplemented diet without any effect on plasma–insulin level. Furthermore, sodium butyrate proved to promote the phosphorylation of Akt, the direct activator of mTOR in IPEC-J2 cell line [49] and in the liver of mice [50], as well as to enhance Akt activation and assembly of rictor (rapamycin insensitive companion of mTOR) and mTOR complex in insulin-resistant HepG2 human cells [50,51]. Although we detected a diminishing effect of exogenous sodium butyrate on IRβ on the protein level and no effect was observed on mTOR on mRNA or protein level, a future investigation of the phosphorylation state of these two insulin-signaling elements could reveal whether butyrate supplementation of the diets altered their activation. Furthermore, the observed interactions highlight that feed additives might exert distinct actions under various dietary conditions; therefore, the complex interplay of different dietary factors should be addressed to improve animal health and productivity using novel nutritional strategies.

Similarly to the observations of GCGR, mTOR and mRNA concentrations were increased in LP groups, but this effect was not realized on a mTOR protein production level. In line with our results, it was reported that a restricted dietary protein supply might affect the expression of certain genes without influencing protein quantities in swine [52,53]. Additionally, the capacity of a low CP diet to alter expression of metabolism-related genes was also detected in studies conducted on broilers [54,55].

## 5. Conclusions

Dietary cereal type remarkably influenced the hepatic endocrine metabolic regulation of broilers in the phase of intensive growth. The wheat-based diet successfully increased the quantity of the investigated members of the insulin signaling pathway on a protein level, with an even more pronounced effect in the NP groups in the case of IRβ. This finding is of outstanding importance, indicating increased hepatic insulin sensitivity, which might be, in certain cases, beneficial due to the growth-promoting effect of insulin. Furthermore, butyrate as a feed additive proved its ability to alleviate hepatic GCGR and IRβ protein abundance, while the WB diet and lowered CP level were able to increase GCGR gene expression on the level of transcription only. The obtained results might contribute to a better understanding of the glycemic control of birds and to the opportunity of improving glucose homeostasis, enhancing the production parameters and welfare of broiler chickens. It can be concluded that dietary factors, particularly the cereal type, play a pivotal role in the modulation of the endocrine regulation of the liver in chickens, serving as a key link between nutrition and metabolic health, but the complex interaction of different dietary factors cannot be neglected. Hence, these factors should be considered as useful tools to improve animal health and productivity, which can be applied to promote sustainable poultry production.

## Figures and Tables

**Table 1:** Ingredients and calculated nutrient composition of experimental broiler starter diets without sodium butyrate supplementation.

Ingredients	Unit	Maize-Based Normal CP	Maize-Based Low CP	Wheat-Based Normal CP	Wheat-Based Low CP
Maize	%	57.60	61.00	0	0
Wheat	%	0	0	54.79	62.60
Solvent extr. soybean meal	%	27.00	28.00	31.00	26.48
PL-68 ^†^	%	6.50	0	3.00	0
Sunflower oil	%	3.50	3.50	6.00	5.30
Wheat bran	%	0	1.72	0	0
Limestone	%	1.70	1.60	1.70	1.70
MCP	%	1.80	2.00	1.70	1.70
Salt (NaCl)	%	0.40	0.40	0.40	0.40
L-lysine hydrochloride	%	0.44	0.58	0.38	0.60
DL-methionine	%	0.43	0.44	0.41	0.45
L-threonine	%	0.09	0.22	0.11	0.26
L-tryptophan	%	0.04	0.04	0	0
Vitamin and mineral premix ^‡^	%	0.50	0.50	0.50	0.50
Axtra XB 201 enzyme ^§^	%			0.015	0.015
Calculated values					
Dry matter	%	89.65	89.32	89.78	89.47
Crude protein	%	22.02	18.65	22.05	18.76
Soluble NSP	Mg/kg	506.88	536.80	5133.82	5865.62
ME	MJ/kg	12.65	12.61	12.63	12.62
Ether extract	%	6.54	6.30	7.49	6.62
Crude fiber	%	2.51	2.74	2.88	2.81
Ash	%	6.97	7.23	7.37	7.42
Lysine	%	1.43	1.43	1.44	1.43
Methionine + Cystine	%	1.07	1.05	1.08	1.07
Threonine	%	0.97	0.94	0.94	0.94
Tryptophan	%	0.23	0.25	0.26	0.24
Arginine	%	1.17	1.24	1.34	1.22
Isoleucine	%	0.74	0.78	0.85	0.78
Leucine	%	1.59	1.68	1.52	1.41
Valine	%	0.83	0.88	0.93	0.86
Total Ca	%	1.15	1.15	1.16	1.14
Total P	%	0.79	0.80	0.82	0.80
Available P	%	0.54	0.53	0.56	0.54

CP: crude protein; MCP: monocalcium phosphate; ME: metabolizable energy; NSP: soluble non-starch polysaccharide. † Protein concentrate, by-product of glutamic acid production from bacterial biomass (KJK-Agroteam Ltd., Dombóvár, Hungary). ‡ Provides per kilogram of diet: vitamin A 12,013 IU; vitamin D3 3875 IU; vitamin K 3.3 mg; vitamin E 46.5 IU; vitamin B1 2.33 mg; vitamin B2 7.44 mg; vitamin B6 3.88 mg, vitamin B12 0.016 mg; calcium pantothenate 13.95 mg; folic acid 1.56 mg; niacin 46.5 mg; choline chloride 504 mg; Fe 60 mg; Mn 100 mg; Cu 12.5 mg; Zn 83 mg; Se 0.42 mg; Co 0.28 mg; I 1.25 mg. § Enzymatic activity in the product (DuPont Animal Nutrition, New Century, KS, USA) 12,200 U/g endo-1,4-beta-xylanase and 1520 U/g endo-1,3(4)-beta-glucanase.

**Table 2:** Ingredients and calculated nutrient composition of experimental broiler grower diets without sodium butyrate supplementation.

Ingredients	Unit	Maize-Based Normal CP	Maize-Based Low CP	Wheat-Based Normal CP	Wheat-Based Low CP
Maize	%	60.71	65.31	0	0
Wheat	%	0	0	61.30	66.56
Solvent extr. soybean meal	%	22.20	24.54	19.31	20.01
PL-68 ^†^	%	8.00	1.00	8.50	2.50
Sunflower oil	%	4.80	4.50	6.70	6.50
Wheat bran	%	0	0	0	0
Limestone	%	1.30	1.20	1.35	1.35
MCP	%	1.35	1.60	1.15	1.15
Salt (NaCl)	%	0.40	0.40	0.40	0.40
L-lysine hydrochloride	%	0.34	0.41	0.38	0.48
DL-methionine	%	0.36	0.37	0.35	0.38
L-threonine	%	0	0.15	0.05	0.16
L-tryptophan	%	0.04	0.02	0	0
Vitamin and mineral premix ^‡^	%	0.50	0.50	0.50	0.50
Axtra XB 201 enzyme ^§^	%			0,015	0,015
Calculated values					
Dry matter	%	89.72	89.34	89.90	89.55
Crude protein	%	21.12	17.85	21.10	17.89
Soluble NSP	Mg/kg	534.25	574.73	5743.81	6236.67
ME	MJ/kg	13.27	13.24	13.24	13.24
Ether extract	%	7.96	7.39	8.45	7.92
Crude fiber	%	2.34	2.48	2.51	2.61
Ash	%	5.78	6.03	6.00	6.13
Lysine	%	1.25	1.22	1.25	1.22
Methionine + Cystine	%	0.96	0.95	0.94	0.95
Threonine	%	0.84	0.84	0.85	0.81
Tryptophan	%	0.21	0.20	0.20	0.21
Arginine	%	1.01	1.11	0.97	1.02
Isoleucine	%	0.65	0.72	0.62	0.65
Leucine	%	1.45	1.58	1.14	1.20
Valine	%	0.74	0.81	0.70	0.74
Total Ca	%	0.92	0.93	0.90	0.90
Total P	%	0.68	0.69	0.71	0.67
Available P	%	0.45	0.45	0.49	0.44

CP: crude protein; MCP: monocalcium phosphate; ME: metabolizable energy; NSP: soluble non-starch polysaccharide. † Protein concentrate, by-product of glutamic acid production from bacterial biomass (KJK-Agroteam Ltd., Dombóvár, Hungary). ‡ Provides per kilogram of diet: vitamin A 12,013 IU; vitamin D3 3875 IU; vitamin K 3.3 mg; vitamin E 46.5 IU; vitamin B1 2.33 mg; vitamin B2 7.44 mg; vitamin B6 3.88 mg, vitamin B12 0.016 mg; calcium pantothenate 13.95 mg; folic acid 1.56 mg; niacin 46.5 mg; choline chloride 504 mg; Fe 60 mg; Mn 100 mg; Cu 12.5 mg; Zn 83 mg; Se 0.42 mg; Co 0.28 mg; I 1.25 mg. § 1Enzymatic activity in the product (DuPont Animal Nutrition, New Century, KS, USA) 12,200 U/g endo-1,4-beta-xylanase and 1520 U/g endo-1,3(4)-beta-glucanase.

**Table 3:** Primer pairs used to test genes of interest.

Gene	Primer	Primer Sequence	Amplicon Size	NCBI Accession	Threshold
GAPDH	Forward (5′-3′)	GGGCACGCCATCACTATCTT	187	NM204305.1	0.03
	Reverse (5′-3′)	TCACAAACATGGGGGCATCA			
GCGR	Forward (5′-3′)	ATCCCGTGGGTTGTTGTGAA	195	NM001101035.1	0.02
	Reverse (5′-3′)	CTTGTAGTCGGTGTAGCGCA			
IRβ	Forward (5′-3′)	CAACCCACACTGGTGGTCAT	134	XM 001233398.5	0.0036
	Reverse (5′-3′)	GCAGCCATCTGGATCATTTCTC			
mTOR	Forward (5′-3′)	GTGGCGATCCTATGGCATGA	276	XM417614.6	0.05
	Reverse (5′-3′)	ACGCCTGAAAACGTGGTAGT			

GAPDH: glyceraldehyde-3-phosphate dehydrogenase (housekeeping gene); GCGR: glucagon receptor; IRβ: insulin receptor β; mTOR: mammalian target of rapamycin. Threshold was calculated by software.

**Table 4:** Representative bands corresponding to the investigated proteins, as obtained by Western blotting.

Parameter	MB NP Ctr	MB NP But	MB LP Ctr	MB LP But	WB NP Ctr	WB NP But	WB LP Ctr	WB LP But
GR	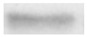	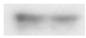	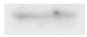	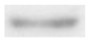	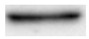	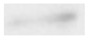	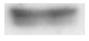	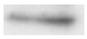
IRβ	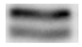	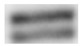	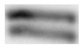	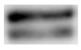	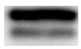	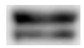	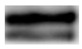	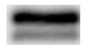
mTOR	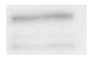	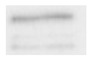	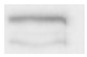	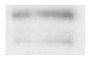	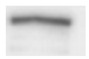	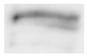	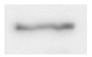	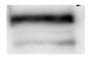

GCGR: glucagon receptor (57 kDal); IRβ: insulin receptor β (95 kDal); mTOR: mammalian target of rapamycin (289 kDal). MB: maize-based diet; WB: wheat-based diet supplemented with NSP-degrading xylanase and glucanase enzymes; NP: “Normal protein” group reared on a diet with crude protein content adequate to the dietary phase; LP: “Low protein” group reared on a diet with crude protein content reduced by 15%, supplemented with limiting amino acids; But: sodium butyrate supplementation of the diet in the dose of 1.5 g/kg diet; Ctr: control group without sodium butyrate supplementation. Western blot Original Images in file S1.

## Data Availability

All raw data supporting the results of the present study can be obtained from the corresponding author upon reasonable request.

## Supplementary Materials

The following supporting information can be downloaded at: https://www.mdpi.com/article/10.3390/vetsci9030103/s1. File S1: Western blot Original Images. Table S1: Linear model coefficient estimates and their standard errors fitted to the gene expression and protein abundance data for each analyzed signaling element. Table S2: ANOVA analysis results of the gene expression and protein abundance data for each analyzed signaling element. Table S3: Post hoc pairwise comparisons of the different treatment groups of the glucagon receptor gene expression data. Table S4: Post hoc pairwise comparisons of the different treatment groups of the glucagon receptor protein abundance data. Table S5: Post hoc pairwise comparisons of the different treatment groups of the insulin receptor β gene expression data. Table S6: Post hoc pairwise comparisons of the different treatment groups of the insulin receptor β protein abundance data. Table S7: Post hoc pairwise comparisons of the different treatment groups of the mammalian target of rapamycin gene expression data. Table S8: Post hoc pairwise comparisons of the different treatment groups of the mammalian target of rapamycin protein abundance data.
