# Supplementary material for: Modulation of Hepatic Insulin and Glucagon Signaling by Nutritional Factors in Broiler Chicken
Source: Vet Sci. 2022 Feb 25;9(3):103. doi: 10.3390/vetsci9030103 (PMC8955576; doi:10.3390/vetsci9030103)
Supplement: Supplementary file 1 [file vetsci-09-00103-s001.zip › vetsci-1608311-supplementary/Table S1-S8.pdf]

Table S1. Linear model coefficient estimates and their standard errors fitted to the gene expression and protein abundance data for each analyzed signaling element. (Petrilla_et_al_Table_S1)

Glucagon receptor				
	Gene expression		Protein abundance	
	Estimate	SE	Estimate	SE
**Intercept**	0.1543	0.0422	1.3519	0.2712
**Cereal-WB**	0.0195	0.0596	1.0107	0.3940
**Protein-LP**	0.0773	0.0581	-0.3828	0.3835
**Butyrate-But**	0.0116	0.0596	-0.5123	0.3835
**Cereal-WB:Protein-LP**	0.1269	0.0832	-0.4942	0.5498
**Cereal-WB:Butyrate-But**	0.0549	0.0832	-0.8763	0.5572
**Protein-LP:Butyrate-But**	-0.0125	0.0822	0.9769	0.5423
**Cereal-WB:Protein-LP:Butyrate-But**	-0.1200	0.1162	-0.0588	0.7775
**Insulin receptor β**				
	Gene expression		Protein abundance	
	Estimate	SE	Estimate	SE
**Intercept**	0.1485	0.0444	0.7394	0.0791
**Cereal-WB**	-0.0808	0.0645	0.7673	0.1118
**Protein-LP**	-0.0163	0.0645	-0.0206	0.1090
**Butyrate-But**	-0.0766	0.0645	-0.2766	0.1090
**Cereal-WB:Protein-LP**	0.2426	0.0924	-0.5054	0.1541
**Cereal-WB:Butyrate-But**	0.2230	0.0924	-0.0714	0.1541
**Protein-LP:Butyrate-But**	0.0791	0.0912	0.2660	0.1521
**Cereal-WB:Protein-LP:Butyrate-But**	-0.4093	0.1317	0.2324	0.2151
**Mammalian target of rapamycin**				
	Gene expression		Protein abundance	
	Estimate	SE	Estimate	SE
**Intercept**	0.0035	0.0013	1.1869	0.4277
**Cereal-WB**	0.0013	0.0017	0.7111	0.6215
**Protein-LP**	0.0037	0.0017	0.1169	0.6049
**Butyrate-But**	0.0015	0.0017	-0.4869	0.6416
**Cereal-WB:Protein-LP**	0.0025	0.0024	0.1240	0.8672
**Cereal-WB:Butyrate-But**	-0.0010	0.0024	0.8517	0.8932
**Protein-LP:Butyrate-But**	-0.0022	0.0024	0.9313	0.8818
**Cereal-WB:Protein-LP:Butyrate-But**	-0.0015	0.0033	-0.5158	1.2368

WB: Wheat based diet supplemented with NSP-degrading xylanase and glucanase enzymes; LP: "Low protein" group reared on a diet with crude protein content reduced by 15 %, supplemented with limiting amino acids; But: Sodium butyrate supplementation of the diet in the dose of 1.5 g/kg diet; SE: Standard Error of the Estimate. Nutritional factors with colon in between show interactions (Cereal: Cereal type; Protein: Crude protein level; Butyrate: Butyrate supplementation). N=10 per group. Model fitting was performed with the *lm* built-in function of the R statistical programming environment (v4.0.3).

Table S2. ANOVA analysis results of the gene expression and protein abundance data for each analyzed signaling element. (Petrilla_et_al_Table_S2)

	Gene expression		Protein abundance	
**Glucagon receptor**				
	**F statistics**	**P value**	**F statistics**	**P value**
**Cereal type**	7.8132	0.0067*	2.4425	0.1226
**Crude protein level**	12.5253	0.0007*	0.5888	0.4455
**Butyrate supplement.**	0.0082	0.9279	5.4513	0.0224*
**Cereal:Protein**	1.2630	0.2650	1.8139	0.1824
**Cereal:Butyrate**	0.0134	0.9083	5.4408	0.0226*
**Protein:Butyrate**	1.5586	0.2162	5.9535	0.0172*
**Cereal:Protein:Butyrate**	1.0672	0.3052	0.0057	0.9399
**Insulin receptor β**				
	**Gene expression**		**Protein abundance**	
	**F statistics**	**P value**	**F statistics**	**P value**
**Cereal type**	2.1432	0.1480	97.7768	<0.0001*
**Crude protein level**	1.7829	0.1865	2.0532	0.1563
**Butyrate supplement.**	0.6197	0.4340	4.6587	0.0343*
**Cereal:Protein**	0.3916	0.5336	12.8919	0.0006*
**Cereal:Butyrate**	0.1074	0.7442	0.1993	0.6567
**Protein:Butyrate**	3.1747	0.0795	12.6278	0.0007*
**Cereal:Protein:Butyrate**	9.6671	0.0028*	1.1677	0.2836
**Mammalian target of rapamycin**				
	**Gene expression**		**Protein abundance**	
	**F statistics**	**P value**	**F statistics**	**P value**
**Cereal type**	4.1528	0.0456*	11.7942	0.0010*
**Crude protein level**	17.4478	<0.0001*	2.6624	0.1073
**Butyrate supplement.**	0.3849	0.5371	0.9219	0.3403
**Cereal:Protein**	1.1052	0.2970	0.0440	0.8346
**Cereal:Butyrate**	1.1016	0.2977	0.8895	0.3489
**Protein:Butyrate**	3.1432	0.0809	1.1711	0.2829
**Cereal:Protein:Butyrate**	0.1997	0.6565	0.1739	0.6780

F statistics and P values are given in the corresponding columns. The study was conducted with two types of cereal (wheat-based diet supplemented with NSP-degrading xylanase and glucanase enzymes [WB] or maize-based diet [MB]), normal (NP) or lowered crude protein level (LP; reduced by 15 %, supplemented with limiting amino acids), and with or without sodium (n-)butyrate supplementation (1.5 g/kg diet). Main effects were determined as follows: WB vs. MB diet (Cereal type), LP vs. NP groups (Crude protein level) and butyrate supplementation vs. no added butyrate (Butyrate supplement.). Nutritional factors with colon in between show interactions (Cereal: Cereal type; Protein: Crude protein level; Butyrate: Butyrate supplement.). N=10 per group. Asterisk (*) indicates statistically significant effects ($P < 0.05$). Calculations were performed with the *Anova* function in the *car* package of the R statistical programming environment (v4.0.3).

Table S3. *Post hoc* pairwise comparisons of the different treatment groups of the glucagon receptor gene expression data. (Petrilla_et_al_Table_S3)

Contrast	Estimate	SE	CI	DF	t value	P value
MB NP Ctr – MB LP Ctr	-0.0773	0.0581	(-0.2589, 0.1044)	68	-1.330	0.8841
MB NP Ctr – WB NP Ctr	-0.0195	0.0596	(-0.2059, 0.1669)	68	-0.327	>0.9999
MB NP Ctr – WB LP Ctr	-0.2236	0.0596	(-0.4100, -0.0372)	68	-3.752	0.0083*
MB NP Ctr – MB NP But	-0.0116	0.0596	(-0.1980, 0.1748)	68	-0.195	>0.9999
MB NP Ctr – MB LP But	-0.0764	0.0581	(-0.2580, 0.1053)	68	-1.315	0.8902
MB NP Ctr – WB NP But	-0.0860	0.0581	(-0.2677, 0.0957)	68	-1.480	0.8150
MB NP Ctr – WB LP But	-0.1576	0.0581	(-0.3393, 0.0241)	68	-2.713	0.1366
MP LP Ctr – WB NP Ctr	0.0578	0.0581	(-0.1239, 0.2394)	68	0.994	0.9738
MP LP Ctr – WB LP Ctr	-0.1464	0.0581	(-0.3280, 0.0353)	68	-2.519	0.2051
MP LP Ctr – MB NP But	0.0656	0.0581	(-0.1160, 0.2473)	68	1.130	0.9479
MP LP Ctr – MB LP But	0.0009	0.0565	(-0.1759, 0.1777)	68	0.016	>0.9999
MP LP Ctr – WB NP But	-0.0087	0.0565	(-0.1856, 0.1681)	68	-0.155	>0.9999
MP LP Ctr – WB LP But	-0.0803	0.0565	(-0.2571, 0.0965)	68	-1.420	0.8447
WB NP Ctr – WB LP Ctr	-0.2041	0.0596	(-0.3905, -0.0177)	68	-3.425	0.0221*
WB NP Ctr – MB NP But	0.0079	0.0596	(-0.1785, 0.1943)	68	0.132	>0.9999
WB NP Ctr – MB LP But	-0.0569	0.0581	(-0.2385, 0.1248)	68	-0.979	0.9759
WB NP Ctr – WB NP But	-0.0665	0.0581	(-0.2482 , 0.1152)	68	-1.145	0.9442
WB NP Ctr – WB LP But	-0.1381	0.0581	(-0.3197, 0.0436)	68	-2.377	0.2694
WB LP Ctr – MB NP But	0.2120	0.0596	(0.0256, 0.3984)	68	3.557	0.0150*
WB LP Ctr – MB LP But	0.1473	0.0581	(-0.0344, 0.3289)	68	2.535	0.1989
WB LP Ctr – WB NP But	0.1376	0.0581	(-0.0440, 0.3193)	68	2.369	0.2733
WB LP Ctr – WB LP But	0.0660	0.0581	(-0.1156, 0.2477)	68	1.137	0.9462
MB NP But – MB LP But	-0.0648	0.0581	(-0.2464, 0.1169)	68	-1.115	0.9514
MB NP But – WB NP But	-0.0744	0.0581	(-0.2561, 0.1073)	68	-1.280	0.9030
MB NP But – WB LP But	-0.1460	0.0581	(-0.3276, 0.0357)	68	-2.513	0.2080
MB LP But – WB NP But	-0.0096	0.0565	(-0.1865, 0.1672)	68	-0.170	>0.9999
MB LP But – WB LP But	-0.0812	0.0565	(-0.2580, 0.0956)	68	-1.436	0.8371
WB NP But – WB LP But	-0.0716	0.0565	(-0.2484, 0.1052)	68	-1.266	0.9081

The calculated difference of the estimated marginal means of the treatment groups compared (Contrast) can be found in the column Estimate, alongside with its standard error and confidence interval (columns SE and CI, respectively). Degrees of freedom (DF), t value and P values can be found in their corresponding columns. Asterisk (*) indicates statistically significant differences ($P < 0.05$). Pairwise comparisons were performed by the *emmeans* package of the R statistical programming environment (v4.0.3), P values and confidence intervals were adjusted with the Tukey method.

Table S4. *Post hoc* pairwise comparisons of the different treatment groups of the glucagon receptor protein abundance data. (Petrilla_et_al_Table_S4)

Contrast	Estimate	SE	CI	DF	t value	P value
MB NP Ctr – MB LP Ctr	0.3828	0.383	(-0.815, 1.581)	70	0.998	0.9732
MB NP Ctr – WB NP Ctr	-1.0107	0.394	(-2.242, 0.220)	70	-2.565	0.1864
MB NP Ctr – WB LP Ctr	-0.1337	0.383	(-1.332, 1.064)	70	-0.349	>0.9999
MB NP Ctr – MB NP But	0.5123	0.383	(-0.686, 1.711)	70	1.336	0.8818
MB NP Ctr – MB LP But	-0.0817	0.383	(-1.280, 1.116)	70	-0.213	>0.9999
MB NP Ctr – WB NP But	0.3779	0.394	(-0.853, 1.609)	70	0.959	0.9786
MB NP Ctr – WB LP But	0.3369	0.383	(-0.861, 1.535)	70	0.878	0.9871
MP LP Ctr – WB NP Ctr	-1.3935	0.394	(-2.625, -0.163)	70	-3.537	0.0157*
MP LP Ctr – WB LP Ctr	-0.5165	0.383	(-1.715, 0.682)	70	-1.347	0.8774
MP LP Ctr – MB NP But	0.1295	0.383	(-1.069, 1.328)	70	0.338	>0.9999
MP LP Ctr – MB LP But	-0.4645	0.383	(-1.663, 0.734)	70	-1.211	0.9259
MP LP Ctr – WB NP But	-0.0049	0.394	(-1.236, 1.226)	70	-0.012	>0.9999
MP LP Ctr – WB LP But	-0.0459	0.383	(-1.244, 1.152)	70	-0.120	>0.9999
WB NP Ctr – WB LP Ctr	0.8770	0.394	(-0.354, 2.108)	70	2.226	0.3494
WB NP Ctr – MB NP But	1.5231	0.394	(0.292, 2.754)	70	3.866	0.0057*

WB NP Ctr – MB LP But	0.9290	0.394	(-0.302, 2.160)	70	2.358	0.2783
WB NP Ctr – WB NP But	1.3886	0.404	(0.126, 2.652)	70	3.435	0.0212*
WB NP Ctr – WB LP But	1.3476	0.394	(0.117, 2.579)	70	3.420	0.0221*
WB LP Ctr – MB NP But	0.6461	0.383	(-0.552, 1.844)	70	1.685	0.6969
WB LP Ctr – MB LP But	0.0520	0.383	(-1.146, 1.250)	70	0.136	>0.9999
WB LP Ctr – WB NP But	0.5116	0.394	(-0.719, 1.743)	70	1.299	0.8964
WB LP Ctr – WB LP But	0.4706	0.383	(-0.728, 1.669)	70	1.227	0.9210
MB NP But – MB LP But	-0.5941	0.383	(-1.792, 0.604)	70	-1.549	0.7780
MB NP But – WB NP But	-0.1344	0.394	(-1.365, 1.097)	70	-0.341	>0.9999
MB NP But – WB LP But	-0.1755	0.383	(-1.374, 1.023)	70	-0.458	0.9998
MB LP But – WB NP But	0.4596	0.394	(-0.771, 1.691)	70	1.167	0.9386
MB LP But – WB LP But	0.4186	0.383	(-0.780, 1.617)	70	1.092	0.9565
WB NP But – WB LP But	-0.0411	0.394	(-1.272, 1.190)	70	-0.104	>0.9999

The calculated difference of the estimated marginal means of the treatment groups compared (Contrast) can be found in the column Estimate, alongside with its standard error and confidence interval (columns SE and CI, respectively). Degrees of freedom (DF), t value and P values can be found in their corresponding columns. Asterisk (*) indicates statistically significant differences ($P < 0.05$). Pairwise comparisons were performed by the *emmeans* package of the R statistical programming environment (v4.0.3), P values and confidence intervals were adjusted with the Tukey method.

Table S5. *Post hoc* pairwise comparisons of the different treatment groups of the insulin receptor β gene expression data. (Petrilla_et_al_Table_S5)

Contrast	Estimate	SE	CI	DF	t value	P value
MB NP Ctr – MB LP Ctr	0.0163	0.0645	(-0.1856, 0.2181)	65	0.252	>0.9999
MB NP Ctr – WB NP Ctr	0.0808	0.0645	(-0.1210, 0.2827)	65	1.254	0.9120
MB NP Ctr – WB LP Ctr	-0.1455	0.0645	(-0.3474, 0.0563)	65	-2.258	0.3326
MB NP Ctr – MB NP But	0.0766	0.0645	(-0.1253, 0.2785)	65	1.189	0.9324
MB NP Ctr – MB LP But	0.0138	0.0627	(-0.1827, 0.2103)	65	0.220	>0.9999
MB NP Ctr – WB NP But	-0.0655	0.0645	(-0.2674, 0.1363)	65	-1.017	0.9702
MB NP Ctr – WB LP But	0.0383	0.0665	(-0.1701, 0.2467)	65	0.576	0.9991
MP LP Ctr – WB NP Ctr	0.0646	0.0661	(-0.1425, 0.2717)	65	0.977	0.9762
MP LP Ctr – WB LP Ctr	-0.1619	0.0661	(-0.3689, 0.0453)	65	-2.446	0.2374
MP LP Ctr – MB NP But	0.0604	0.0661	(-0.1467, 0.2675)	65	0.913	0.9838
MP LP Ctr – MB LP But	-0.0025	0.0645	(-0.2043, 0.1994)	65	-0.038	>0.9999
MP LP Ctr – WB NP But	-0.0818	0.0661	(-0.2889, 0.1253)	65	-1.237	0.9177
MP LP Ctr – WB LP But	0.0221	0.0682	(-0.1914, 0.2356)	65	0.324	>0.9999
WB NP Ctr – WB LP Ctr	-0.2264	0.0661	(-0.4335, -0.0192)	65	-3.423	0.0226*
WB NP Ctr – MB NP But	-0.0042	0.0661	(-0.2113, 0.2029)	65	-0.064	>0.9999
WB NP Ctr – MB LP But	-0.0671	0.0645	(-0.2689, 0.1348)	65	-1.040	0.9663
WB NP Ctr – WB NP But	-0.1464	0.0661	(-0.3535, 0.0607)	65	-2.213	0.3577
WB NP Ctr – WB LP But	-0.0425	0.0682	(-0.2560, 0.1710)	65	-0.624	0.9984
WB LP Ctr – MB NP But	0.2222	0.0661	(0.0150, 0.4293)	65	3.359	0.0270*
WB LP Ctr – MB LP But	0.1593	0.0645	(-0.0426, 0.3612)	65	2.472	0.2261
WB LP Ctr – WB NP But	0.0800	0.0661	(-0.1271, 0.2871)	65	1.210	0.9262
WB LP Ctr – WB LP But	0.1839	0.0682	(-0.0296, 0.3973)	65	2.697	0.1421
MB NP But – MB LP But	-0.0628	0.0645	(-0.2647, 0.1390)	65	-0.975	0.9765
MB NP But – WB NP But	-0.1422	0.0661	(-0.3493, 0.0650)	65	-2.150	0.3954
MB NP But – WB LP But	-0.0383	0.0682	(-0.2518, 0.1752)	65	-0.562	0.9992
MB LP But – WB NP But	-0.0793	0.0645	(-0.2812, 0.1225)	65	-1.231	0.9197
MB LP But – WB LP But	0.0245	0.0665	(-0.1839, 0.2329)	65	0.369	>0.9999
WB NP But – WB LP But	0.1039	0.0682	(-0.1096, 0.3174)	65	1.524	0.7919

The calculated difference of the estimated marginal means of the treatment groups compared (Contrast) can be found in the column Estimate, alongside with its standard error and confidence interval (columns SE and CI, respectively). Degrees of freedom (DF), t value and P values can be found in their corresponding columns. Asterisk (*) indicates statistically significant differences ($P < 0.05$). Pairwise comparisons were performed by the *emmeans* package of the R statistical programming environment (v4.0.3), P values and confidence intervals were adjusted with the Tukey method.

Table S6. *Post hoc* pairwise comparisons of the different treatment groups of the insulin receptor β protein abundance data. (Petrilla_et_al_Table_S6)

Contrast	Estimate	SE	CI	DF	t value	P value
MB NP Ctr – MB LP Ctr	0.0206	0.109	(-0.3199, 0.3611)	70	0.189	>0.9999
MB NP Ctr – WB NP Ctr	-0.7673	0.112	(-1.1167, -0.4180)	70	-6.862	<.0001*
MB NP Ctr – WB LP Ctr	-0.2414	0.109	(-0.5819, 0.0992)	70	-2.215	0.3560
MB NP Ctr – MB NP But	0.2766	0.109	(-0.0639, 0.6171)	70	2.538	0.1972
MB NP Ctr – MB LP But	0.0312	0.109	(-0.3093, 0.3718)	70	0.287	>0.9999
MB NP Ctr – WB NP But	-0.4194	0.109	(-0.7599, -0.0788)	70	-3.848	0.0060*
MB NP Ctr – WB LP But	-0.3918	0.109	(-0.7324, -0.0513)	70	-3.595	0.0132*
MP LP Ctr – WB NP Ctr	-0.7879	0.109	(-1.1285, -0.4474)	70	-7.229	<.0001*
MP LP Ctr – WB LP Ctr	-0.2620	0.106	(-0.5934, 0.0695)	70	-2.469	0.2258
MP LP Ctr – MB NP But	0.2560	0.106	(-0.0755, 0.5875)	70	2.413	0.2514
MP LP Ctr – MB LP But	0.0106	0.106	(-0.3208, 0.3421)	70	0.100	>0.9999
MP LP Ctr – WB NP But	-0.4400	0.106	(-0.7714, -0.1085)	70	-4.147	0.0023*
MP LP Ctr – WB LP But	-0.4124	0.106	(-0.7439, -0.0809)	70	-3.887	0.0053*
WB NP Ctr – WB LP Ctr	0.5260	0.109	(0.1854, 0.8665)	70	4.826	0.0002*
WB NP Ctr – MB NP But	1.0439	0.109	(0.7034, 1.3845)	70	9.578	<.0001*
WB NP Ctr – MB LP But	0.7986	0.109	(0.4580, 1.1391)	70	7.327	<.0001*
WB NP Ctr – WB NP But	0.3480	0.109	(0.0074, 0.6885)	70	3.193	0.0417*
WB NP Ctr – WB LP But	0.3755	0.109	(0.0350, 0.7161)	70	3.445	0.0206*
WB LP Ctr – MB NP But	0.5180	0.106	(0.1865, 0.8494)	70	4.883	0.0002*
WB LP Ctr – MB LP But	0.2726	0.106	(-0.0589, 0.6041)	70	2.570	0.1848
WB LP Ctr – WB NP But	-0.1780	0.106	(-0.5095, 0.1535)	70	-1.678	0.7011
WB LP Ctr – WB LP But	-0.1504	0.106	(-0.4819, 0.1810)	70	-1.418	0.8459
MB NP But – MB LP But	-0.2454	0.106	(-0.5768, 0.0861)	70	-2.313	0.3015
MB NP But – WB NP But	-0.6960	0.106	(-1.0274, -0.3645)	70	-6.561	<.0001*
MB NP But – WB LP But	-0.6684	0.106	(-0.9999, -0.3369)	70	-6.301	<.0001*
MB LP But – WB NP But	-0.4506	0.106	(-0.7821, -0.1192)	70	-4.248	0.0016*
MB LP But – WB LP But	-0.4230	0.106	(-0.7545, -0.0916)	70	-3.988	0.0038*
WB NP But – WB LP But	0.0276	0.106	(-0.3039, 0.3590)	70	0.260	>0.9999

The calculated difference of the estimated marginal means of the treatment groups compared (Contrast) can be found in the column Estimate, alongside with its standard error and confidence interval (columns SE and CI, respectively). Degrees of freedom (DF), t value and P values can be found in their corresponding columns. Asterisk (*) indicates statistically significant differences ($P < 0.05$). Pairwise comparisons were performed by the *emmeans* package of the R statistical programming environment (v4.0.3), P values and confidence intervals were adjusted with the Tukey method.

Table S7. *Post hoc* pairwise comparisons of the different treatment groups of the mammalian target of rapamycin gene expression data. (Petrilla_et_al_Table_S7)

Contrast	Estimate	SE	CI	DF	t value	P value
MB NP Ctr – MB LP Ctr	-0.0037	0.00174	(-0.0092, 0.0017)	66	-2.146	0.3977
MB NP Ctr – WB NP Ctr	-0.0013	0.00174	(-0.0068, 0.0041)	66	-0.756	0.9947
MB NP Ctr – WB LP Ctr	-0.0076	0.00174	(-0.0130, -0.0021)	66	-4.360	0.0012*
MB NP Ctr – MB NP But	-0.0015	0.00174	(-0.0069, 0.0040)	66	-0.860	0.9885
MB NP Ctr – MB LP But	-0.0030	0.00170	(-0.0084, 0.0023)	66	-1.789	0.6296
MB NP Ctr – WB NP But	-0.0018	0.00170	(-0.0071, 0.0035)	66	-1.076	0.9597
MB NP Ctr – WB LP But	-0.0044	0.00170	(-0.0097, 0.0009)	66	-2.600	0.1745
MP LP Ctr – WB NP Ctr	0.0024	0.00169	(-0.0029, 0.0077)	66	1.433	0.8387
MP LP Ctr – WB LP Ctr	-0.0039	0.00169	(-0.0091, 0.0014)	66	-2.283	0.3185
MP LP Ctr – MB NP But	0.0022	0.00169	(-0.0030, 0.0075)	66	1.325	0.8860
MP LP Ctr – MB LP But	0.0007	0.00165	(-0.0045, 0.0058)	66	0.422	0.9999
MP LP Ctr – WB NP But	0.0019	0.00165	(-0.0032, 0.0071)	66	1.158	0.9406

MP LP Ctr – WB LP But	-0.0007	0.00165	(-0.0058, 0.0045)	66	-0.415	0.9999
WB NP Ctr – WB LP Ctr	-0.0063	0.00169	(-0.0116, -0.0010)	66	-3.716	0.0094*
WB NP Ctr – MB NP But	-0.0002	0.00169	(-0.0055, 0.0051)	66	-0.108	>0.9999
WB NP Ctr – MB LP But	-0.0017	0.00165	(-0.0069, 0.0034)	66	-1.048	0.9650
WB NP Ctr – WB NP But	-0.0005	0.00165	(-0.0057, 0.0046)	66	-0.312	>0.9999
WB NP Ctr – WB LP But	-0.0031	0.00165	(-0.0083, 0.0020)	66	-1.885	0.5657
WB LP Ctr – MB NP But	0.0061	0.00169	(0.0008, 0.0114)	66	3.608	0.0131*
WB LP Ctr – MB LP But	0.0045	0.00165	(-0.0006, 0.0097)	66	2.764	0.1221
WB LP Ctr – WB NP But	0.0058	0.00165	(0.0006, 0.0109)	66	3.501	0.0179*
WB LP Ctr – WB LP But	0.0032	0.00165	(-0.0020, 0.0083)	66	1.927	0.5379
MB NP But – MB LP But	-0.0015	0.00165	(-0.0067, 0.0036)	66	-0.937	0.9811
MB NP But – WB NP But	-0.0003	0.00165	(-0.0055, 0.0048)	66	-0.201	>0.9999
MB NP But – WB LP But	-0.0029	0.00165	(-0.0081, 0.0022)	66	-1.775	0.6391
MB LP But – WB NP But	0.0012	0.00160	(-0.0038, 0.0062)	66	0.757	0.9947
MB LP But – WB LP But	-0.0014	0.00160	(-0.0064, 0.0036)	66	-0.860	0.9885
WB NP But – WB LP But	-0.0026	0.00160	(-0.0076, 0.0024)	66	-1.617	0.7386

The calculated difference of the estimated marginal means of the treatment groups compared (Contrast) can be found in the column Estimate, alongside with its standard error and confidence interval (columns SE and CI, respectively). Degrees of freedom (DF), t value and P values can be found in their corresponding columns. Asterisk (*) indicates statistically significant differences (P < 0.05). Pairwise comparisons were performed by the *emmeans* package of the R statistical programming environment (v4.0.3), P values and confidence intervals were adjusted with the Tukey method.

Table S8. *Post hoc* pairwise comparisons of the different treatment groups of the mammalian target of rapamycin protein abundance data. (Petrilla_et_al_Table_S8)

Contrast	Estimate	SE	CI	DF	t value	P value
MB NP Ctr – MB LP Ctr	-0.117	0.605	(-2.008, 1.774)	69	-0.193	>0.9999
MB NP Ctr – WB NP Ctr	-0.711	0.621	(-2.654, 1.232)	69	-1.144	0.9444
MB NP Ctr – WB LP Ctr	-0.952	0.605	(-2.843, 0.939)	69	-1.574	0.7640
MB NP Ctr – MB NP But	0.487	0.642	(-1.519, 2.492)	69	0.759	0.9946
MB NP Ctr – MB LP But	-0.561	0.605	(-2.452, 1.330)	69	-0.928	0.9823
MB NP Ctr – WB NP But	-1.076	0.605	(-2.967, 0.815)	69	-1.779	0.6363
MB NP Ctr – WB LP But	-1.732	0.605	(-3.623, 0.159)	69	-2.864	0.0963
MP LP Ctr – WB NP Ctr	-0.594	0.621	(-2.537, 1.348)	69	-0.956	0.9790
MP LP Ctr – WB LP Ctr	-0.835	0.605	(-2.726, 1.056)	69	-1.380	0.8630
MP LP Ctr – MB NP But	0.604	0.642	(-1.402, 2.609)	69	0.941	0.9808
MP LP Ctr – MB LP But	-0.444	0.605	(-2.335, 1.446)	69	-0.735	0.9956
MP LP Ctr – WB NP But	-0.959	0.605	(-2.850, 0.932)	69	-1.585	0.7572
MP LP Ctr – WB LP But	-1.615	0.605	(-3.506, 0.275)	69	-2.671	0.1495
WB NP Ctr – WB LP Ctr	-0.241	0.621	(-2.183, 1.702)	69	-0.388	0.9999
WB NP Ctr – MB NP But	1.198	0.657	(-0.856, 3.252)	69	1.823	0.6071
WB NP Ctr – MB LP But	0.150	0.621	(-1.793, 2.092)	69	0.241	>0.9999
WB NP Ctr – WB NP But	-0.365	0.621	(-2.307, 1.578)	69	-0.587	0.9989
WB NP Ctr – WB LP But	-1.021	0.621	(-2.964, 0.921)	69	-1.643	0.7227
WB LP Ctr – MB NP But	1.439	0.642	(-0.567, 3.444)	69	2.243	0.3402
WB LP Ctr – MB LP But	0.391	0.605	(-1.500, 2.281)	69	0.646	0.9980
WB LP Ctr – WB NP But	-0.124	0.605	(-2.015, 1.767)	69	-0.205	>0.9999
WB LP Ctr – WB LP But	-0.780	0.605	(-2.671, 1.110)	69	-1.290	0.8995
MB NP But – MB LP But	-1.048	0.642	(-3.054, 0.957)	69	-1.634	0.7284
MB NP But – WB NP But	-1.563	0.642	(-3.568, 0.443)	69	-2.436	0.2411
MB NP But – WB LP But	-2.219	0.642	(-4.225, -0.214)	69	-3.459	0.0199*
MB LP But – WB NP But	-0.515	0.605	(-2.405, 1.376)	69	-0.851	0.9893
MB LP But – WB LP But	-1.171	0.605	(-3.062, 0.720)	69	-1.936	0.5318
WB NP But – WB LP But	-0.656	0.605	(-2.547, 1.234)	69	-1.085	0.9579

The calculated difference of the estimated marginal means of the treatment groups compared (Contrast) can be found in the column Estimate, alongside with its standard error and confidence interval (columns SE and CI, respectively). Degrees of freedom (DF), t value and P values can be found in their corresponding columns. Asterisk (*) indicates statistically

significant differences ($P < 0.05$). Pairwise comparisons were performed by the *emmeans* package of the R statistical programming environment (v4.0.3), P values and confidence intervals were adjusted with the Tukey method.
