# Supplementary figures and images for: Modulation of Hepatic Insulin and Glucagon Signaling by Nutritional Factors in Broiler Chicken
Source: Vet Sci. 2022 Feb 25;9(3):103. doi: 10.3390/vetsci9030103 (PMC8955576; doi:10.3390/vetsci9030103)

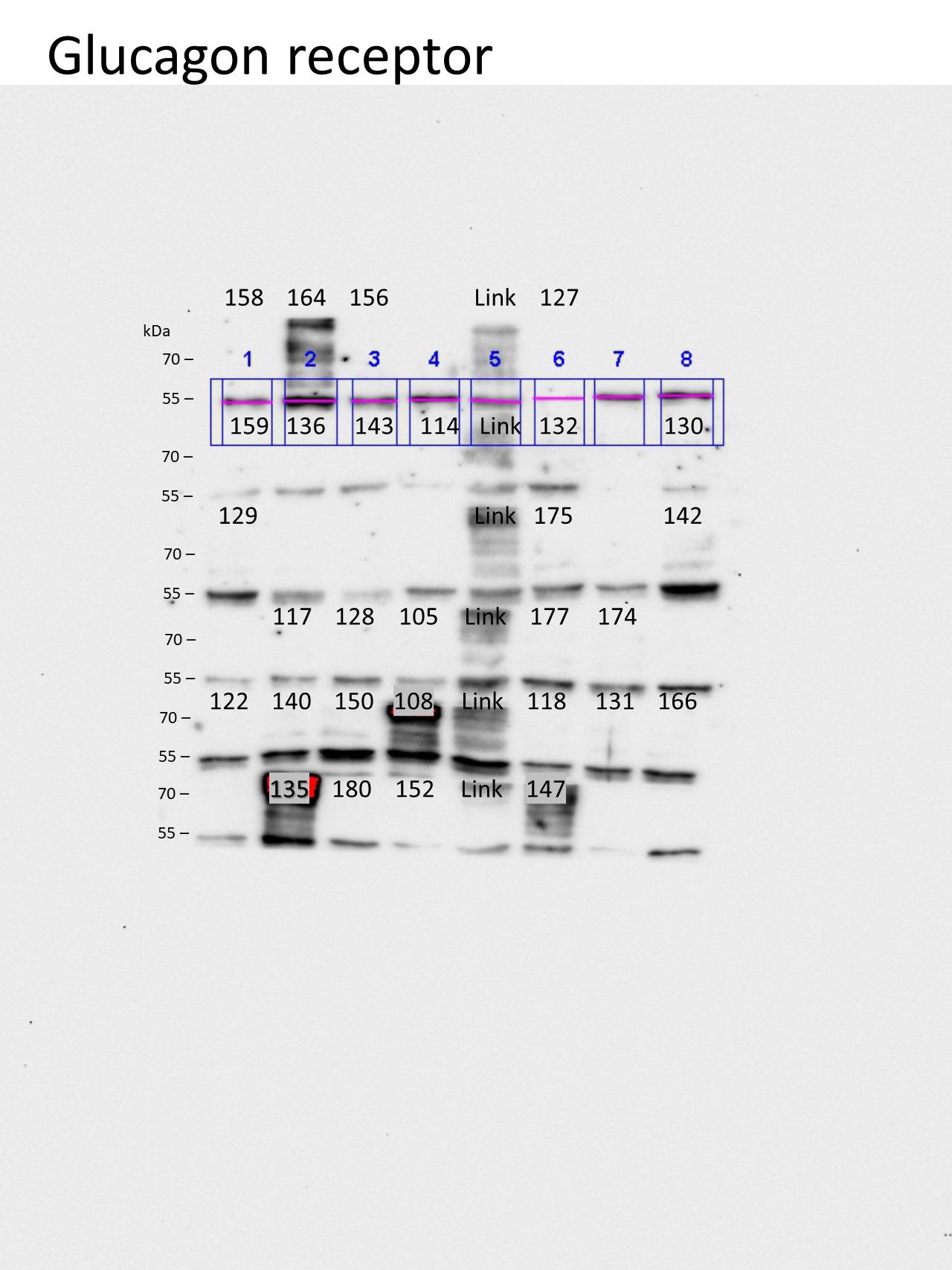

Supplement: Supplementary file 1 [file vetsci-09-00103-s001.zip › vetsci-1608311-supplementary/File S1/Glucagon_receptor_1.JPG]

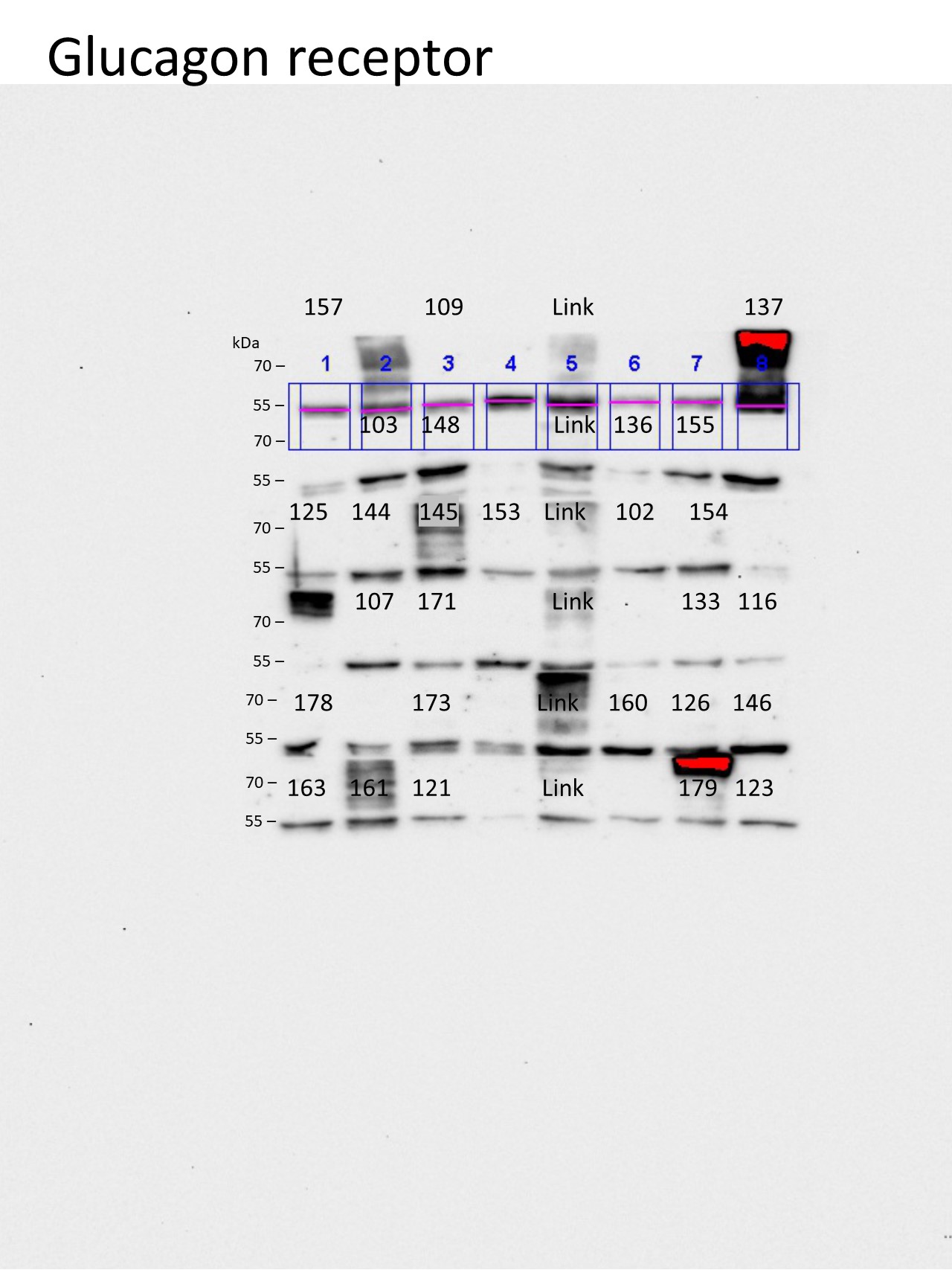

Supplement: Supplementary file 1 [file vetsci-09-00103-s001.zip › vetsci-1608311-supplementary/File S1/Glucagon_receptor_2.JPG]

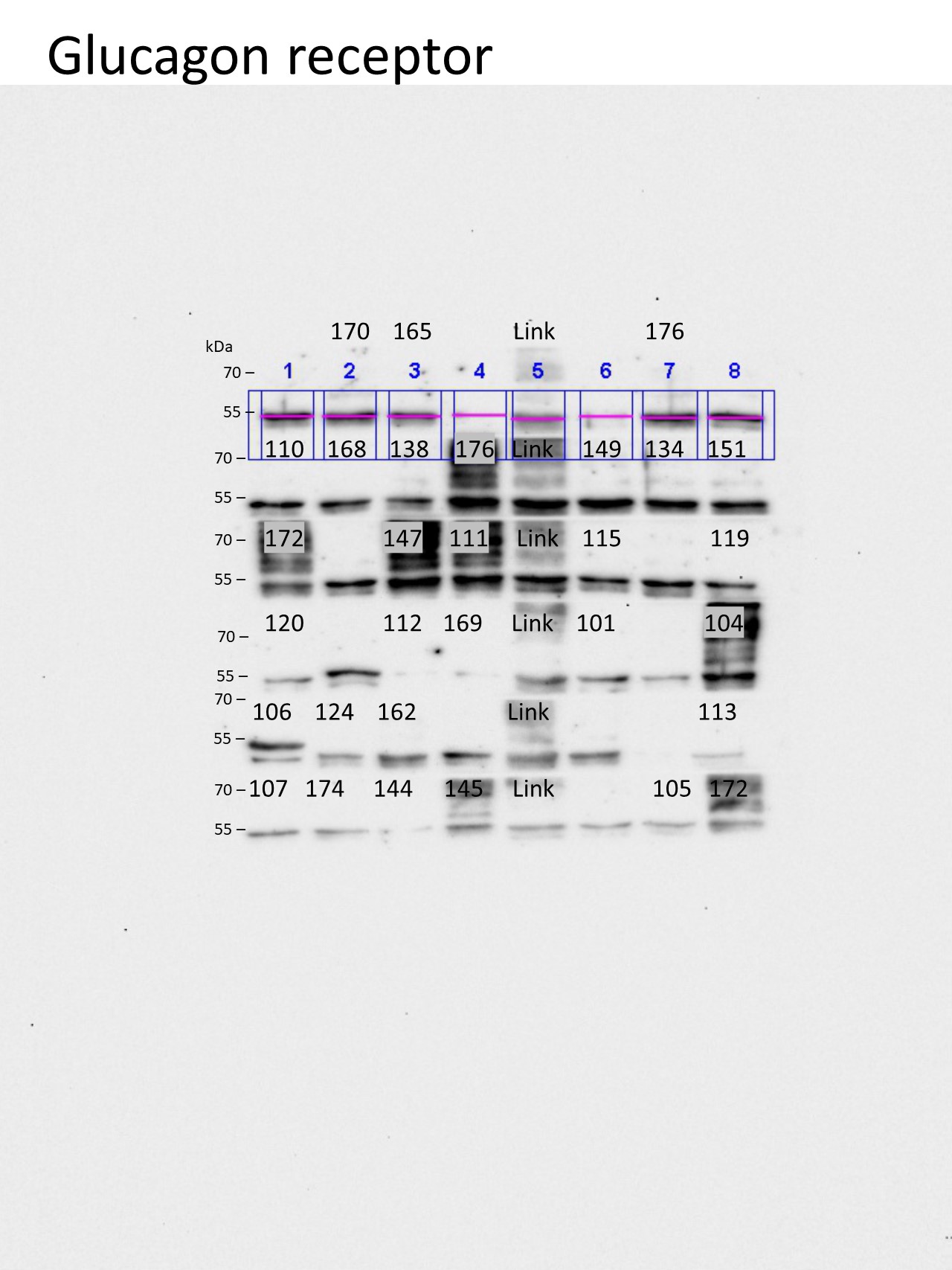

Supplement: Supplementary file 1 [file vetsci-09-00103-s001.zip › vetsci-1608311-supplementary/File S1/Glucagon_receptor_3.JPG]

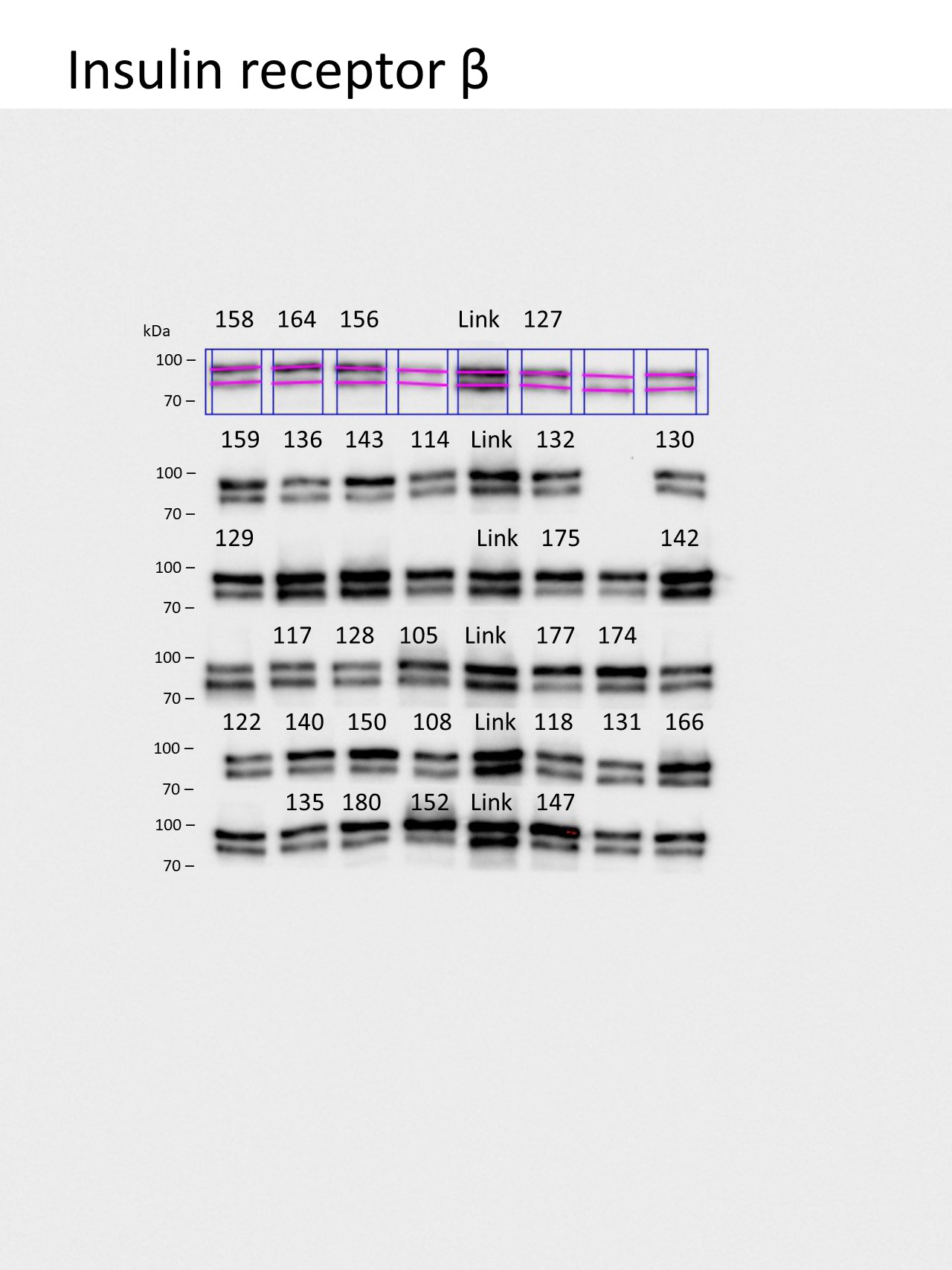

Supplement: Supplementary file 1 [file vetsci-09-00103-s001.zip › vetsci-1608311-supplementary/File S1/Insulin_receptor_beta_1.JPG]

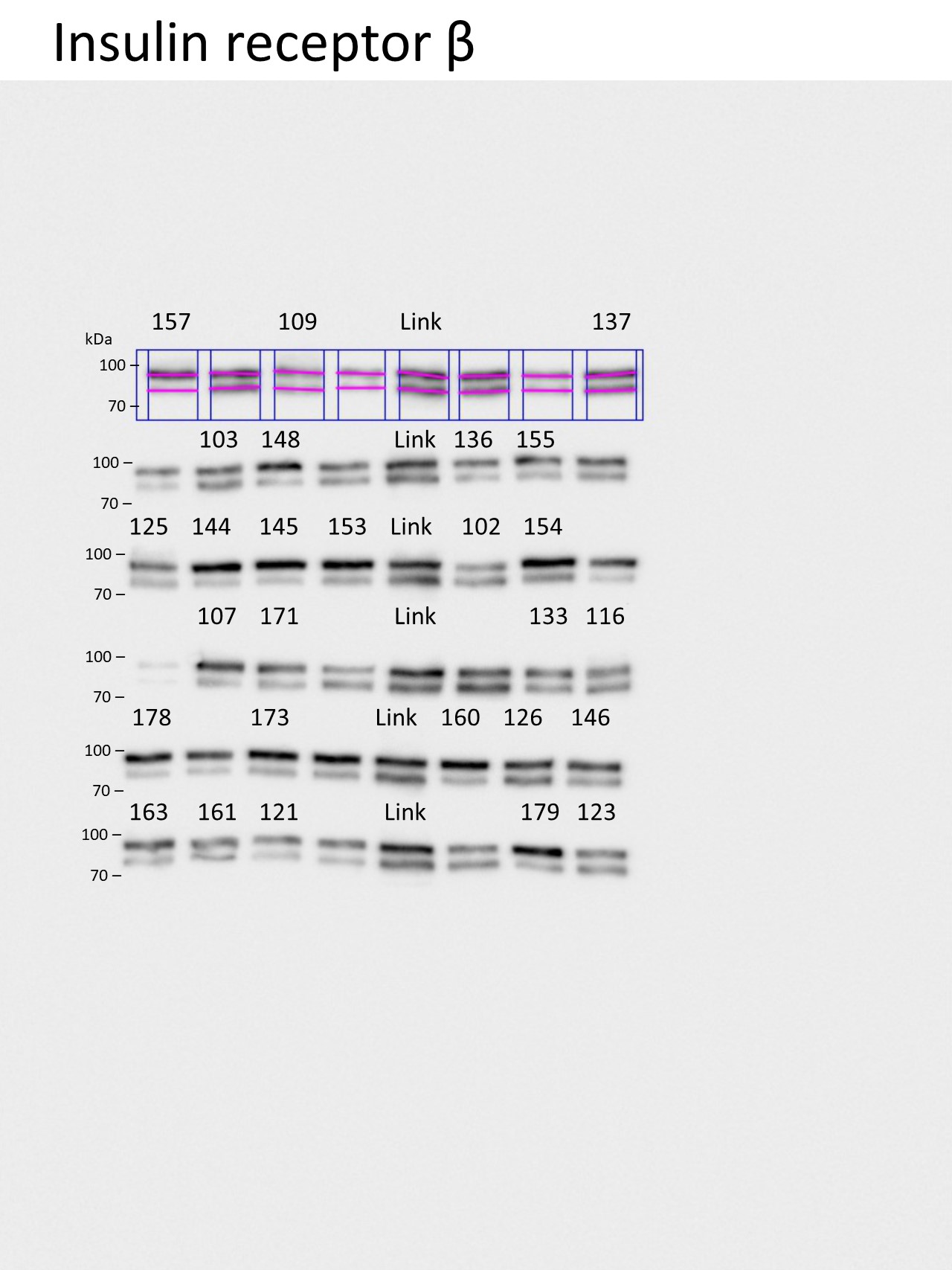

Supplement: Supplementary file 1 [file vetsci-09-00103-s001.zip › vetsci-1608311-supplementary/File S1/Insulin_receptor_beta_2.JPG]

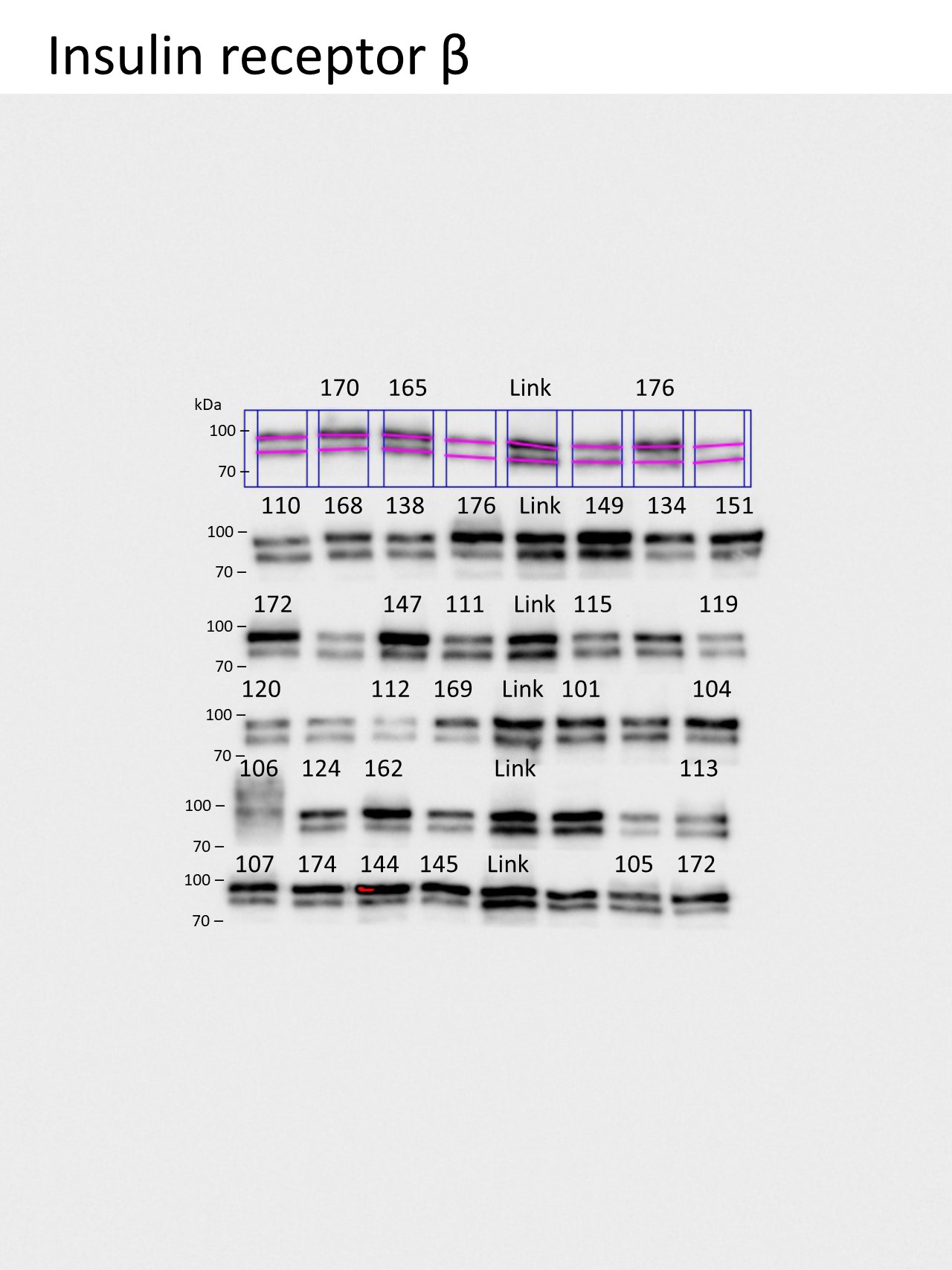

Supplement: Supplementary file 1 [file vetsci-09-00103-s001.zip › vetsci-1608311-supplementary/File S1/Insulin_receptor_beta_3.JPG]

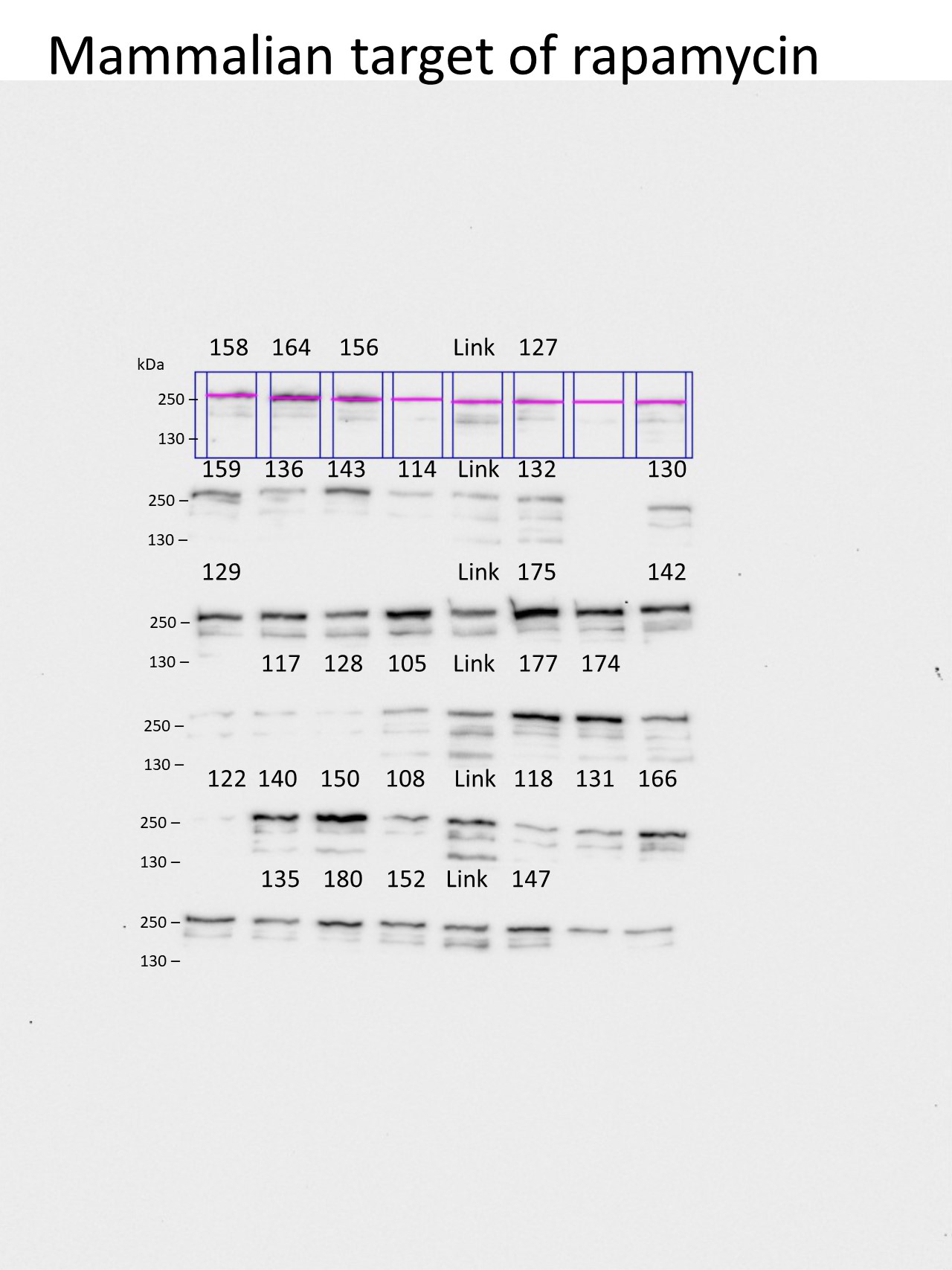

Supplement: Supplementary file 1 [file vetsci-09-00103-s001.zip › vetsci-1608311-supplementary/File S1/Mammalian_target_of_rapamycin_1.JPG]

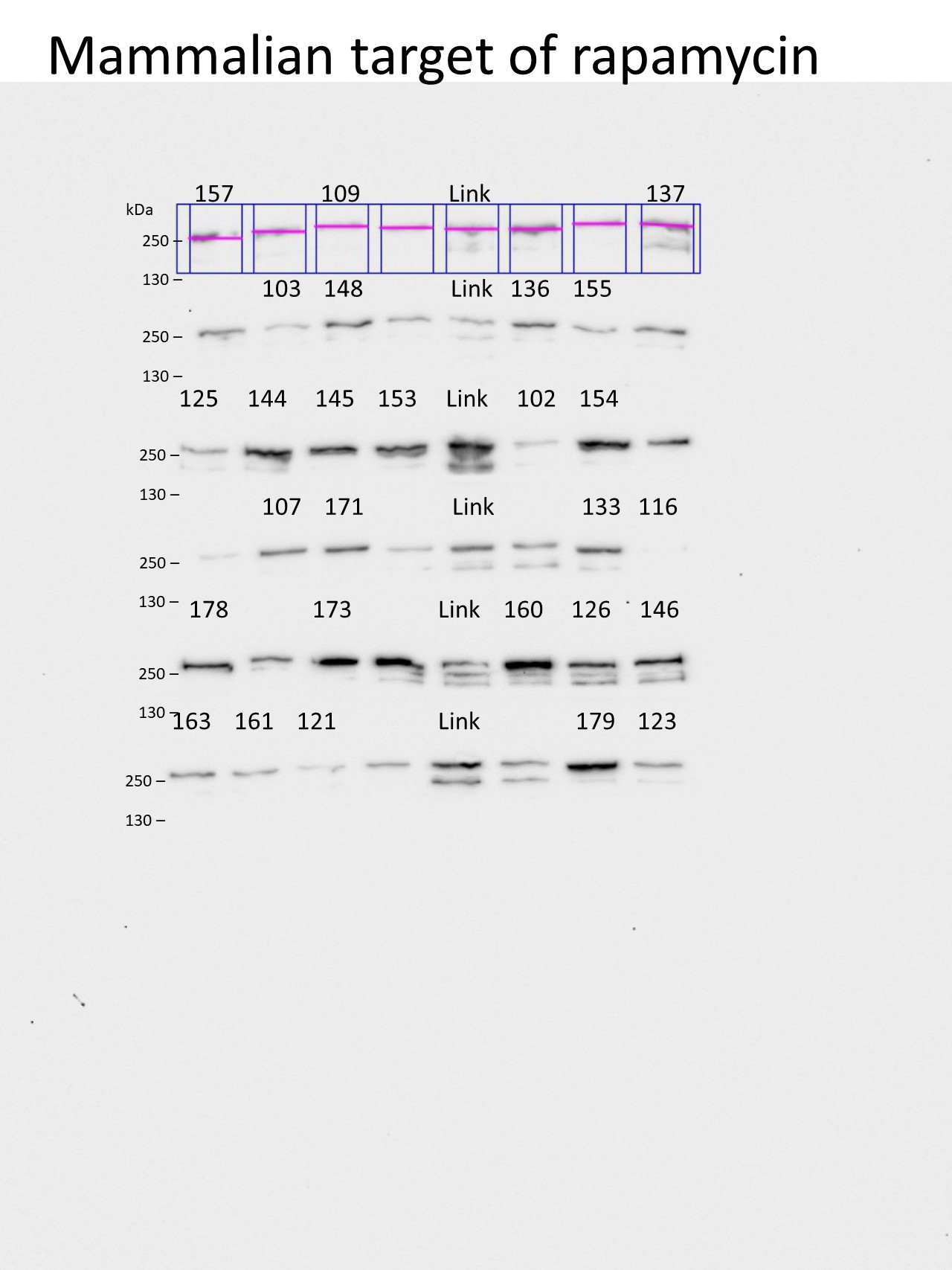

Supplement: Supplementary file 1 [file vetsci-09-00103-s001.zip › vetsci-1608311-supplementary/File S1/Mammalian_target_of_rapamycin_2.JPG]

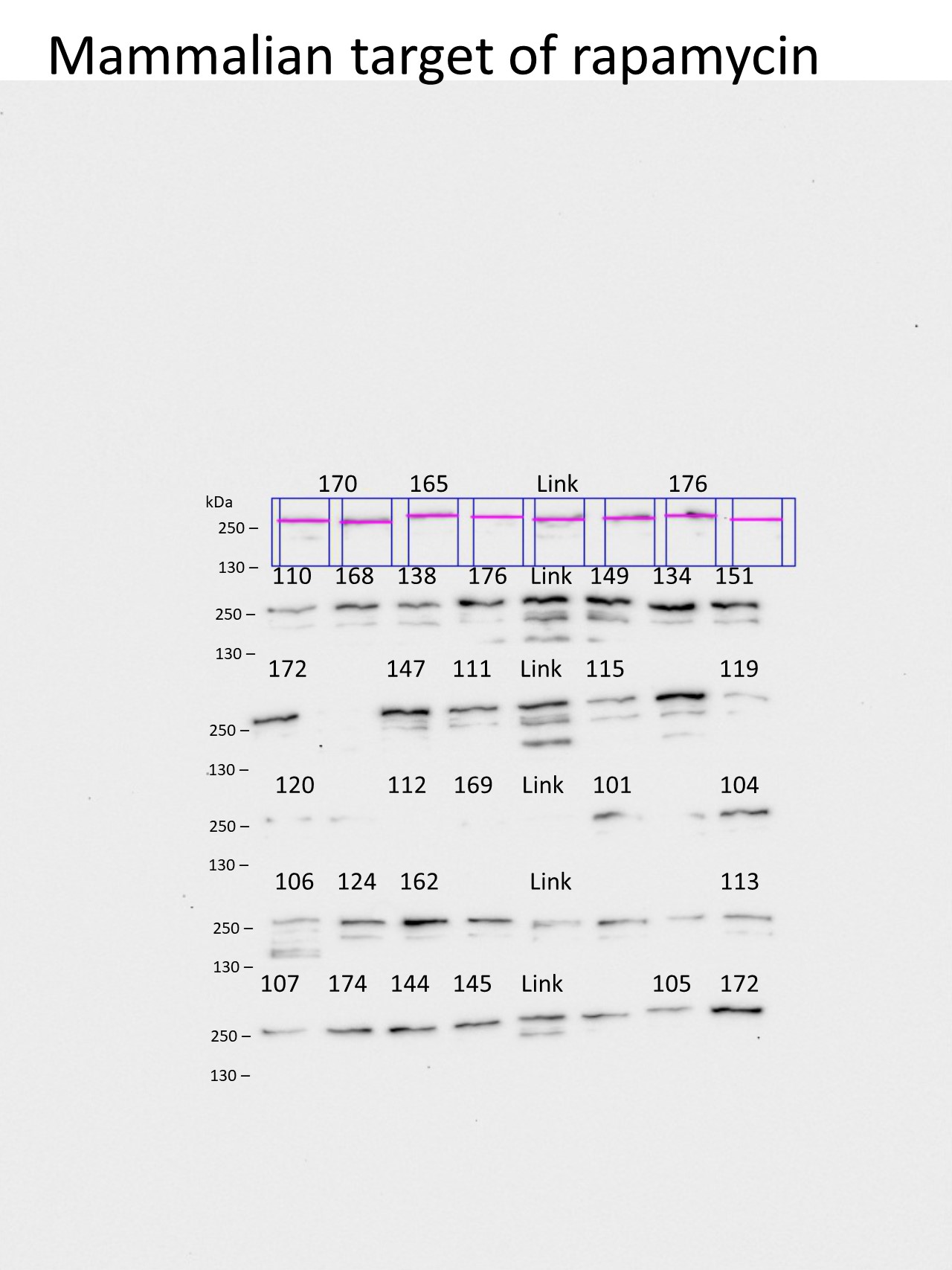

Supplement: Supplementary file 1 [file vetsci-09-00103-s001.zip › vetsci-1608311-supplementary/File S1/Mammalian_target_of_rapamycin_3.JPG]
